# The complexity of tracking stegosaurs and their gregarious behavior

**DOI:** 10.1038/s41598-024-64298-9

**Published:** 2024-07-03

**Authors:** Diego Castanera, Luis Mampel, Alberto Cobos

**Affiliations:** Fundación Conjunto Paleontológico de Teruel-Dinópolis/Museo Aragonés de Paleontología, Avenida de Sagunto s/n, 44002 Teruel, Spain

**Keywords:** Palaeontology, Behavioural ecology

## Abstract

Stegosaur tracks were unknown until the identification of *Deltapodus* more than 20 years ago. Currently, the Iberian Peninsula, especially Teruel Province, is one of the areas globally with the most occurrences of these tracks. However, their identification, based on the global record, is problematic due to their similarities with sauropod tracks. A review of the largest number of analyzed *Deltapodus* tracks globally, including the holotype of *D. ibericus* and a description of new occurrences, has been carried out. Our research shows substantial morphological variations, but all the studied tracks can be considered *D. ibericus* based on the manus morphology and the morphometric data. These variations are related to substrate differences and/or different dynamic foot postures (possibly ontogenetically related) during locomotion, as evidenced by changes within the same trackway. We provide detailed comparisons via 3D modeling with sauropod tracks, and our data show that they generally have proportionally longer manus and wider pes because of the differences in the metapodial bones. The scarcity of stegosaur trackways in the fossil record has prevented the identification of gregarious behavior in this group of herbivorous dinosaurs. Two of the studied tracksites show evidence of this behavior, being the only examples among stegosaurs described thus far in the fossil record.

## Introduction

Stegosauria is a clade of thyreophoran ornithischian dinosaurs characterized by the presence of two rows of dorsal dermal plates and spines that extend from the neck to the end of the tail and, from a locomotive point of view, by their quadrupedality^1^. Since the beginning of dinosaur ichnology (see^2^ and references therein), stegosaur tracks were not properly known until the identification of *Deltapodus* as a stegosaur track^3,4^. *Deltapodus* is characterized by impressions with a characteristic subtriangular pes with three short, rounded, blunt digits in the anterior position, as well as a crescentic manus^3–6^. The type ichnospecies *D. brodricki* was originally described from Middle Jurassic strata in England^3,4^. Since then, *Deltapodus* tracks have been described with confidence in several areas that span the Middle Jurassic–Early Cretaceous interval in Europe (e.g.,^5,7–10^ and references therein), America (e.g.,^11–13^), Africa^14^ and Asia^6,15^.

In the last decade, researchers have reported several localities with *Deltapodus* tracks on the Iberian Peninsula, which is one of the areas with the most track occurrences globally. Thus, *Deltapodus* is currently a commonly identified ichnotaxon in the Late Jurassic and Jurassic‒Cretaceous transitional units, with significant tracksites in the Maestrazgo^5,16–20^, Cameros^8,21^ and Asturian^22–24^ Basins in Spain and in the Lusitanian Basin^7,10^ in Portugal. The presence of *Deltapodus* tracks and their attribution to stegosaur trackmakers in the Iberian Peninsula is reinforced by the presence of ample stegosaur osteological remains in Late Jurassic deposits of the Iberian Peninsula (e.g., ^5,25–28^ and references therein).

Despite the characteristic features of *Deltapodus* tracks, their attribution to stegosaur trackmakers can be a challenge, especially when evaluating poorly preserved tracks. This problem with the identification of the trackmaker of *Deltapodus* tracks has recurred since the original description of *D. brodricki*, which was originally thought to be produced by a sauropod and posteriorly reinterpreted as thyreophor in origin^3,4^. Similarly, the type locality of *D. ibericus* [the El Castellar 1 (CT-1) tracksite] was originally thought to be sauropod-dominated until the identification of the holotype trackway [see^5^ and references therein]. Some footprints from the Maestrazgo Basin (AG-3 tracksite) have also been interpreted either as stegosaur^16^ or sauropod^29^ in origin due to the difficulties in the identification of both groups of dinosaurs, despite their anatomical differences^2,4,5^.

Gregarious behavior has been identified in several groups of dinosaurs by studying ichnological and osteological records^30^. Notably, whereas this type of behavior is common in sauropods^30–32^, among stegosaurs, it has been hypothesized only for the CT-1^18^ and AG-3^16^ tracksites, but their evidence has not been described in detail.

Recently, new *Deltapodus* tracks were discovered at two new sites (LPV-1 and FA-11 in the municipalities of La Puebla de Valverde and Formiche Alto) in the Maestrazgo Basin. In addition, several specimens from tracksites from the El Castellar municipality, including part of the CT-1 tracksite, have not been described in detail^5,18^. Thus, the aims of the present study are multifold: (1) to describe these new specimens; (2) to review previously described material in the Maestrazgo Basin assigned to *Deltapodus*; (3) to analyze footprint variability and discuss the causes of the variation and ichnotaxonomic implications; (4) to compare the material with sauropod tracks and provide clues for discriminating between the two groups of quadrupedal dinosaurs; and (5) to infer paleoecological data (possible gregarious behavior) among stegosaur trackmakers.

## Geographical and geological setting

**Figure 1:**
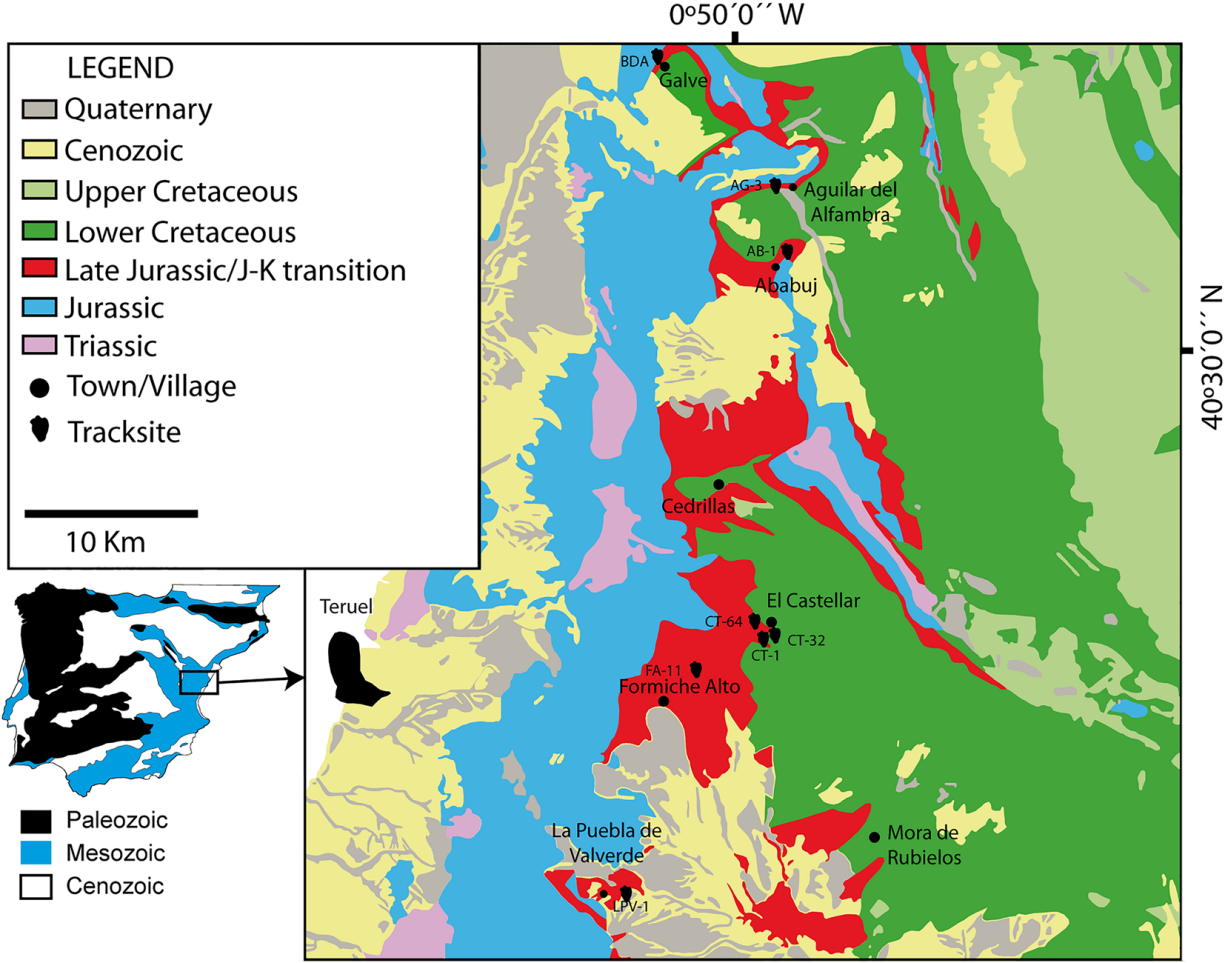
Geographical and geological setting of the tracksites with *Deltapodus* tracks in the Maestrazgo Basin (Teruel Province). The simplified geological map was redrawn from^34^.

The studied tracks originate from different Late Jurassic tracksites (see Table S1) in the Teruel Province. They are located in the Maestrazgo Basin (see Fig. 1) in both the Peñagolosa and Galve subbasins, which are the westernmost subbasins of the Iberian Basin Rift System^19,33–35^ and references therein]. In the Peñagolosa subbasin, *Deltapodus* tracks have been identified in the El Castellar municipality at the El Castellar 1 (CT-1), La Balsa (CT-32) and CT-64 tracksites^5,18,19^. Recently, new *Deltapodus* tracks have been discovered at the LPV-1 and FA-11 tracksites, which are located in the La Puebla de Valverde and Formiche Alto municipalities, respectively. Three tracksites in the Galve subbasin yielded Late Jurassic *Deltapodus*-like tracks. These tracksites are located in the municipalities of Galve^20^ (Barranco del Agua, BDA), Aguilar del Alfambra^16^ (Aguilar 3, AG-3) and Ababuj^17^ (AB-1). All these tracksites are situated (see Table S1) in the Villar del Arzobispo Formation (*sensu*^19,34^), which has been correlated with the Cedrillas and Aguilar del Alfambra Formations (*sensu* ^33,35,36^). The ages of the Villar del Arzobispo Fm. in the Peñagolosa subbasin are constrained by large benthic foraminifera as Kimmeridgian-Tithonian^19,34^. In the Galve subbasin, the ages of these deposits range from late Kimmeridgian to early Berriasian and are constrained by the presence of large benthic foraminifera, charophytes, ostracods and strontium isotope data^33,35–37^.

Most of the tracks are preserved as true tracks (concave epireliefs) on top of limestone beds, whereas others are preserved as casts (either convex epireliefs or hyporeliefs) in sandstone beds. This variety of modes of preservation is related to the different depositional conditions of each tracksite (Table S1), which vary from sediments deposited in a coastal and alluvial plain to inter- to supratidal conditions in a tidal flat environment or muddy coastal plain related to a wave-dominated delta and a carbonate lagoon^18,19,33–37^.

## Results

**Figure 2:**
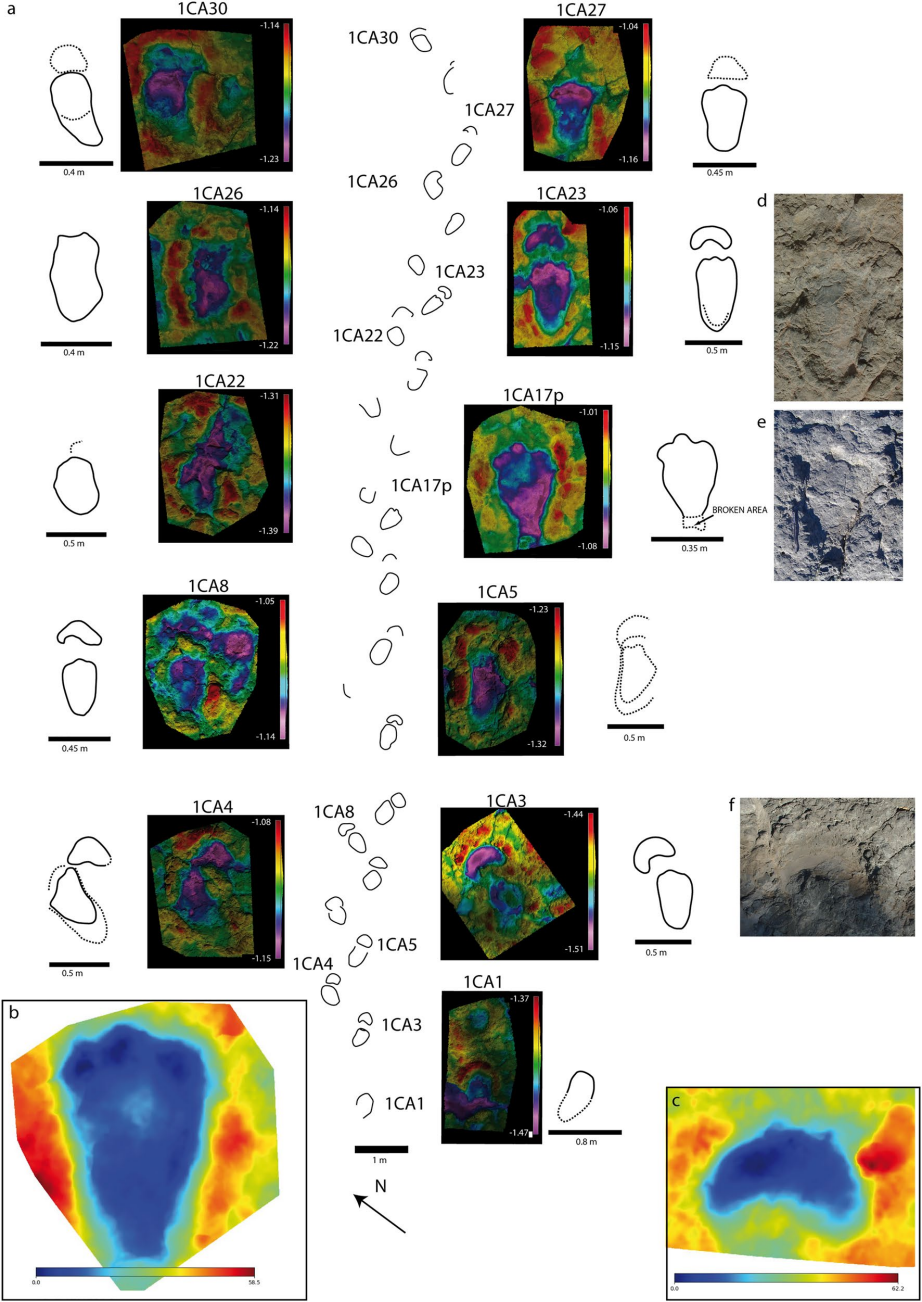
Morphological variation in the *Deltapodus ibericus* holotype trackway from the CT-1 tracksite. (**a**) False-color depth maps and interpretative outline drawings of a selection of manus–pes sets within the trackway. Note the variation in manus (from crescent, kidney to semicircular shapes) and pes (reversed delta, semirectangular, and oval shapes) tracks depending on the manus–pes set. Note also that some manus are overprinted by the pes. (**b**) Mediotype^68^ of the pes impressions based on 4 specimens with a mean footprint length of 0.48 m. Note that the morphology of the posterior part of the print is slightly biased (more quadrangular) because this area in track 1CA17p is broken. (**c**) Mediotype^68^ of the manus impressions based on 2 specimens with a mean footprint width of 0.37 m. (**d–f**) Pictures of a selection of the best-preserved tracks (manus–pes set 1CA23, pes 1CA17p and manus 1CA3m). Fig. S1 shows the location of the trackway within the CT-1 tracksite. The false color depth maps were generated with the software CloudCompare (https://www.cloudcompare.org/) and the mediotypes with the software DigTrace (https://www.digtrace.co.uk/). See scaled 3D models in the Supplementary Information.

**Figure 3:**
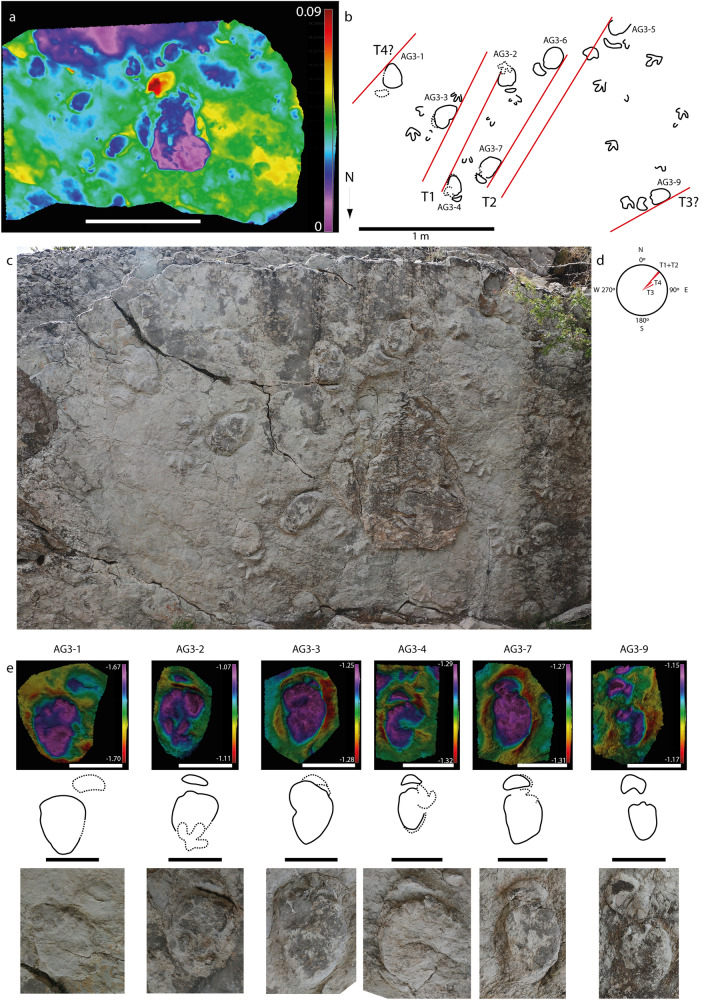
*Deltapodus* tracks from the Aguilar 3 (AG-3) tracksite. (**a**) False-color depth map of part of the main surface of the AG-3 tracksite. (**b**) Sketch of the area, emphasizing the *Deltapodus* trackways. Note that only those tracks that can be clearly identified in the 3D model have been drawn. (**c**) Picture of the main surface of the AG-3 tracksite. (**d**) Rose diagram with the orientation of the *Deltapodus* trackways. **e)** False-color depth map, interpretative outline drawings and pictures of selected manus–pes sets. Note that tracks AG3-2 and AG3-4 are overprinted by a tridactyl track. The latter could be interpreted as a sauropod track with laterally oriented claw impressions, but rounded DIII and DIV can be clearly identified in the *Deltapodus* pes. Additionally, note the morphological variation among the manus–pes sets in trackway 1 (T1) and among the manus prints between the different tracks. Additionally, note the manus print in AG3-4, with possible evidence of medially oriented pollex mark impressions. Scale bars = 1 m (a), 0.2 m (d). The false color depth maps were generated with the software CloudCompare (https://www.cloudcompare.org/). See scaled 3D models in the Supplementary Information.

**Figure 4:**
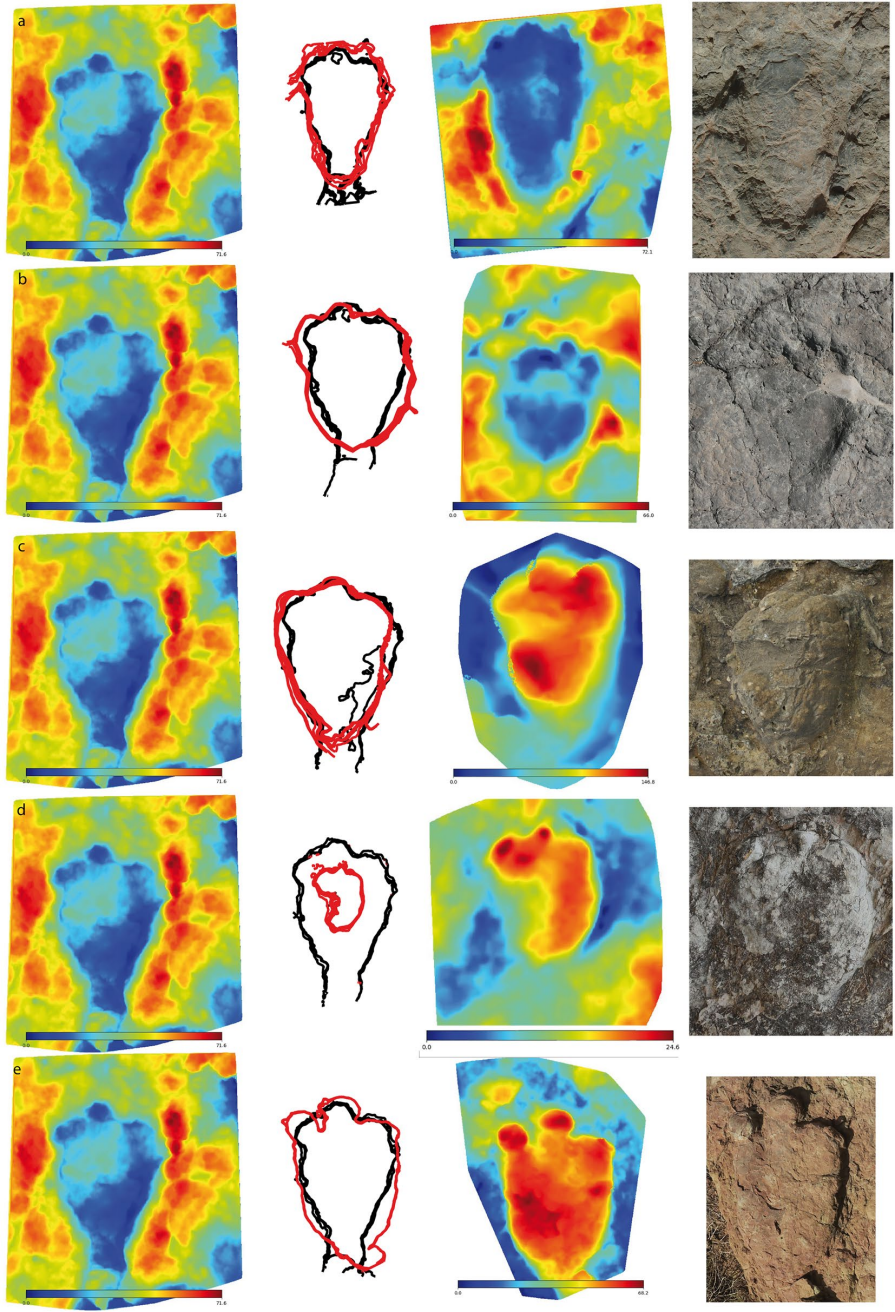
Comparison of the outlines of *Deltapodus* pes constructed with DigTrace software. Track 1CA17p (holotype trackway, see Fig. 2e) was selected as the reference track (left column) and was compared with (**a**) 1CA23p (holotype trackway, see also Fig. 2d), (**b**) 2EC208, (**c**) BDA2-2p, (**d**) AG3-9p and (**e**) LPV-1-3R-1p. The right column shows a picture of each individual track. Note that the tracks are not scaled, and the differences between blue and red represent the preservation of the tracks as true tracks and casts, respectively. Color depth maps were also generated with the software DigTrace (https://www.digtrace.co.uk/). See scaled 3D models in the Supplementary Information.

Detailed descriptions of the footprints and the tracksites are provided in the supplemental information. The studied tracks are characterized by the main features of *Deltapodus*, such as three blunt-toed pes and kidney-shaped manus impressions. Nonetheless, the sample shows morphological changes in both the manus and pes. The pes tracks vary from subtriangular (reversed delta) to semirectangular (depending on the lateral/medial development of the posterior part of the footprint, especially the length and width of the heel pad impression) and oval (depending on the impression of the hoof-like unguals). Variations in the pes shape between these morphologies can be observed among the same and different tracksites (Figs. 2 and 3, S1-S7) and even in the same trackway (Figs. 2 and 3). A comparison of the outlines among the pes prints (Fig. 4) shows that the main variations are in the elongated (with larger heel pad impressions) or more rounded tracks. An interesting feature seen in the different pes tracks in the *D. ibericus* holotype trackway and in other specimens is a lower development in the impression of the lateral digit (DIV) in comparison with the central (DIII) and the medial (DII) digits, which shows that the hoof-like ungual impression has not been preserved in the lateral digit, whereas in the other two, it is clearly visible (see Figs. 2 and 3, S2, S5).

**Figure 5:**
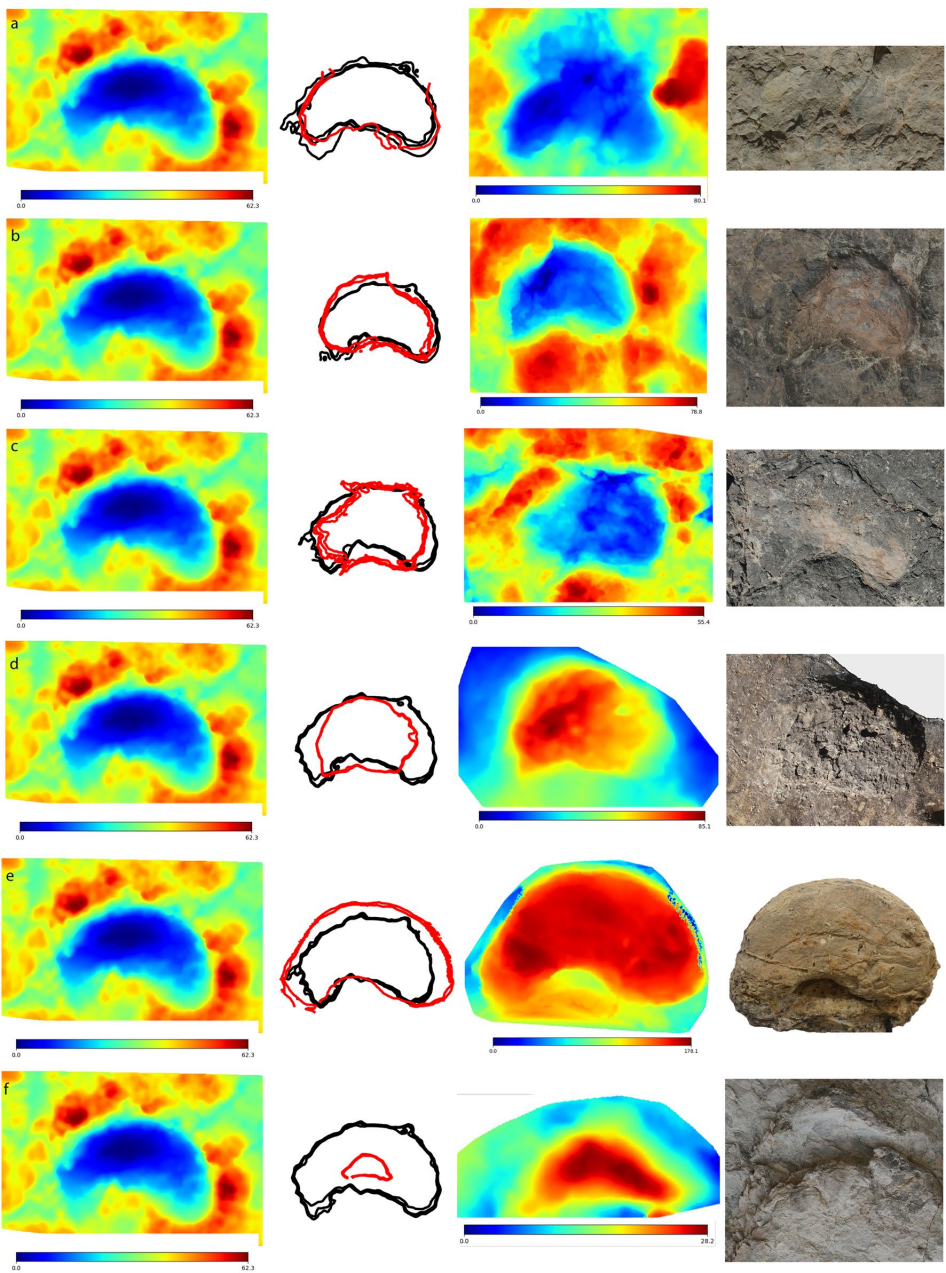
Comparison of the outlines of *Deltapodus* manus built with DigTrace software. Track 1CA3m (holotype trackway, see Fig. 2f) was selected as the reference track (left column) and was compared with (**a**) 1CA23m (holotype trackway), (**b**) 2EC25m, (**c**) 2EC5.1m, (**d**) BDA2-1m (associated with *Deltapodus* pes, see Fig. S6), (**e**) BDA9m, and (**f**) AG3-4m (associated with *Deltapodus* pes, see Fig. 3e). Note that the tracks are not to scale, and the differences between blue and red represent the preservation of the tracks as true tracks and casts, respectively. Color depth maps were also generated with the software DigTrace  (https://www.digtrace.co.uk/). See scaled 3D models in the Supplementary Information.

The morphology of the manus prints vary from kidney to crescent to semicircular/oval (Figs. 2 and 3, S1–S6). A comparison of the outlines of a selection of manus prints (Fig. 5) clearly illustrates these variations, which mainly show major/minor antero-posterior development. A morphological feature not observed in most of the samples is pollex mark impressions. Among the 49 studied manus prints, only AG3-4 and 2EC265m (Figs. 3, S1) show morphologies in the posterior part of the manus that might represent this impression.

The sample shows substantial variability in track size (Figs. S8,S9), i.e., from small (Pes length = 0.15–0.24 m) to medium and large (Pes length = 0.35–0.56 m) tracks in the largest specimens (see Table S2). Many of the specimens are in the medium-sized category, except for some pes impressions in the CT-1 tracksite, which are pes specimens with considerable elongation (Fig. 2). This size variation ranges between the values described for ichnogenus *Deltapodus*^7,8,10,15^.

Analysis of the proportions (Footprint length/width ratio, FL/FW ratio) in both the manus and pes also reveals substantial variability (Table S2). In trackway 1CA at tracksite CT-1 (Fig. 2), the ratio varies for the manus (0.36–0.58) and pes (1.57–1.92). In the AG-3 tracksite (the second largest subsample, Fig. 3), the tracks also show substantial variation in this parameter (0.27–0.55 in the manus and 1.35–1.72 in the pes). This large variation means that the data of the other tracks in the sample overlap between the values of the two tracksites. Interestingly, many of the analyzed manus tracks (25/39, 64%) have FL/FW ratios within the range of those of trackway 1CA (Table S2). In contrast, the majority of the pes specimens (16/23, 69%) have lower FL/FW ratios than do the holotype trackway. A bivariate graph (Fig. S10) showing the FL/FW ratio in both the manus and pes of the available manus–pes sets with high morphological preservation shows that the main differences within the proportions in the sample are in the pes tracks, with those in the holotype of *D. ibericus* (and some specimens in CT-1 and AG-3) being more elongated than those in most of the specimens, including other pes prints from the CT-1 tracksite. The graph also shows the variation in the manus morphology from crescent (lower values) to kidney–semicircular (higher values). The variation in both the manus and pes has a direct influence on heteropody (Table S2), which mainly ranges (excluding those tracks with extramorphological influence) from low (1:2) to intermediate (1:3–1:4). Considering the morphological variation, stat-tracks, including the mediotype of the holotype of *D. ibericus*, are constructed (Fig. 2b,c) to better characterize this ichnospecies.

## Discussion

The studied Maestrazgo Basin sample is worthy of ichnotaxonomic analysis to understand whether the new and previously unclassified (at the ichnospecies level) specimens might belong to the Iberian ichnospecies *D. ibericus* or to the other described ichnospecies. Currently, three different ichnospecies are considered valid within *Deltapodus*: *D. brodricki*, the type ichnospecies described in the Middle Jurassic strata of England^3,4^, the aforementioned *D. ibericus* analyzed here in the Late Jurassic strata of Spain^5^ and *D. curriei* in the Lower Cretaceous strata of China^6,15^. Since the description of the holotype trackway 1CA at the El Castellar (CT-1) tracksite, it has been the only tracksite where this ichnospecies has been identified, and some of the posteriorly described specimens have been classified only at the ichnogenus level (see Table 1 in^10^) in some cases^e.g.,20^, assuming that several of the diagnostic characteristics of *D. ibericus* are based on trackway data. Although some trackway parameters are indeed diagnostic, the ichnospecies *D. ibericus* is also based on characteristic features in both the manus and pes (see the diagnosis in^5^).

**Figure 6:**
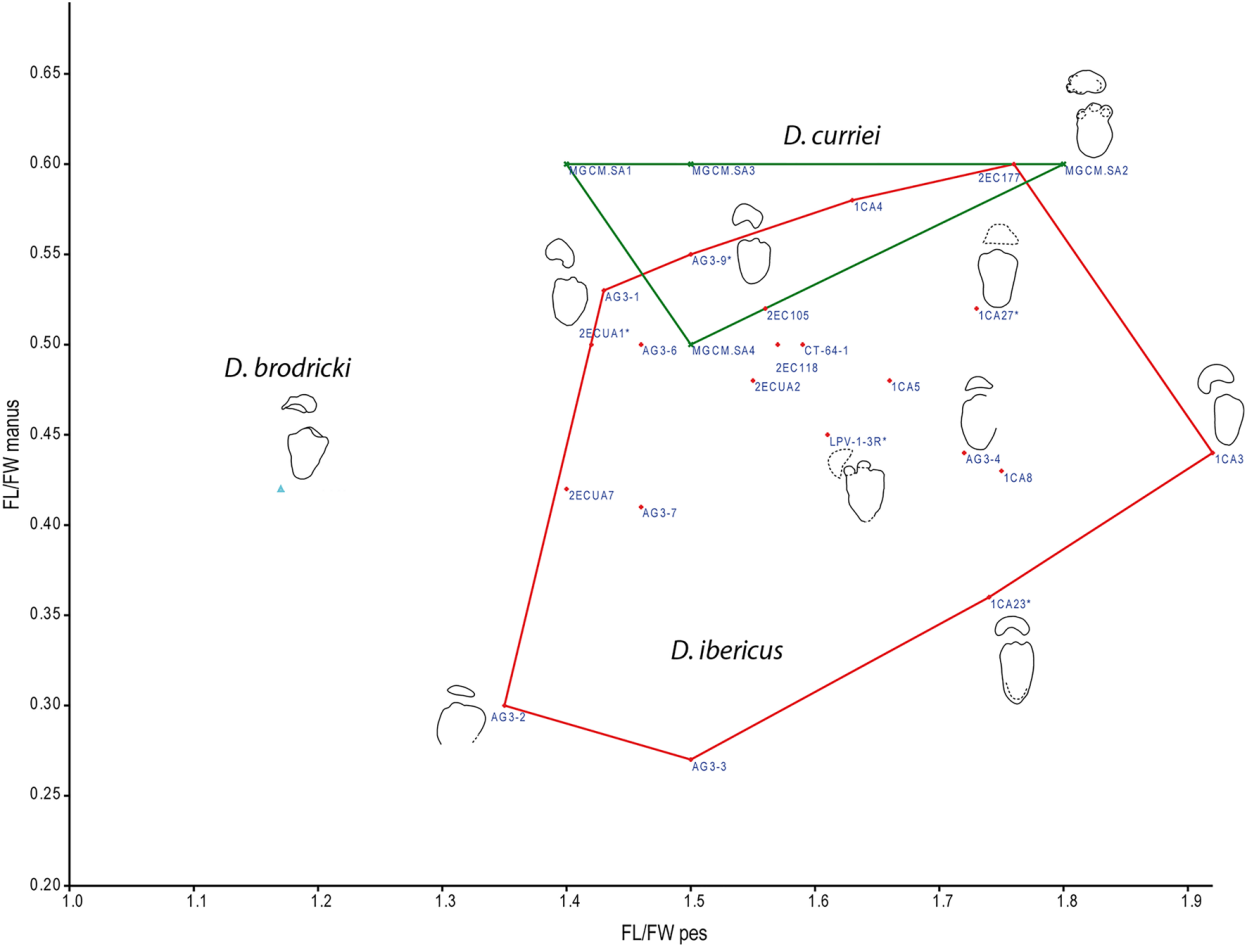
Variation in proportions among *Deltapodus* ichnospecies. Bivariate graph showing the variation in proportions (FL/FW ratio) in both the manus and pes of a selection of manus–pes sets in the studied *Deltapodus ibericus* sample compared with the other two *Deltapodus* ichnospecies (*D. brodricki* and *D. curriei*). Note that the outline drawings have been scaled to a similar size. Graph generated with the software PAST.

Many of the features described for *D. ibericus* are clearly different from those of the type ichnospecies *D. brodricki* (Fig. 6), especially the manus morphology, which is entaxonic, irregular and crescentic with evidence of pollex marks^3,4^. In addition, the main difference between *D. ibericus* and *D. curriei* is also the manus morphology. In the latter case, there is evidence of a clear pollex mark but also the impression of digit II, which is a diagnostic characteristic of this ichnospecies^6^. The overall morphology of the pes differs from that of *D. brodricki*; the pes is triangular and slightly longer than it is wide in the UK ichnospecies^3,4^ and more elongated in *D. ibericus*. The pes shapes of *D. ibericus* and *D. curriei* are very similar. The differences observed between the hoof-like impressions in our sample have also been described in other *Deltapodus* samples^6,15,23^, including *D. curriei*. Color depth maps of the individual pes prints (Figs. 2–3; S1–S7) do not show a considerable difference in depth between the medial and central digits with the lateral digit, so there are no data to suggest that this feature might be influenced by a greater weight in the inner part of the pes during locomotion. Indeed, it may represent an anatomical characteristic of trackmaker feet since some stegosaur pes skeletons seem to show size differences between the unguals^5,38^.

Although the studied sample shows considerable variation in both the manus and pes, the following should be considered: (1) the manus, when better preserved, are kidney-shaped with pollex mark impressions that are absent or reduced and are considerably different from the other two *Deltapodus* ichnospecies. (2) The other manus print morphologies (crescent and semicircular) in the sample are considered extramorphological variations, where the manus leaves a smaller/larger impression of the posterior part, which is sometimes related to a deeper penetration of the forelimb in the substrate and/or influenced by the pes modifying the manus print. (3) The variation in pes morphologies (more rounded or elongated) and in the FL/FW ratio in the pes sample might represent an ichnotaxonomic difference. Nonetheless, these are also considered extramorphological variations, since although the tracks of the holotype trackway show high variation, several tracks from the trackway show values close to those of other tracks from the different tracksites (including from different size classes), and the outlines of these footprints are very similar (Fig. 5).

An analysis of these parameters that compares our sample (Fig. 6) with the other *Deltapodus* ichnospecies also reveals the importance of the pes proportions with more or less elongated footprints. Notably, the parameters of *D. brodricki* are considerably different from those of *D. ibericus* and *D. curriei*, which are more similar. Thus, considering the variation in our sample (Fig. S10), the similarities in the comparisons of the outlines (Figs. 4 and 5), and the fact that the major differences between the ichnospecies are diagnostic features of the manus prints of *D. brodricki* (presence of pollex marks and an entaxonic shape) and *D. curriei* (presence of pollex marks and a characteristic digit II impression), we conclude that there is no argument for considering the studied tracks different from those of the ichnospecies *D. ibericus*; thus, all the studied samples are tentatively included in this ichnospecies. In addition, although some differences between the three ichnospecies can be taphonomic (e.g., *D. brodricki* and *D. curriei* are preserved as casts) and the differences in locomotion/gait and substrate conditions between the tracksites could have affected them, the main differences between the three ichnospecies seem to be primarily anatomical (presence/absence and development of pollex marks and digit II in the manus and larger/shorter development of the pes print). These differences could be related to the different trackmakers, considering the different stegosaur faunas present during the Middle and Late Jurassic in Europe (candidate trackmakers of *D. brodricki*^4^ and *D. ibericus*^5^, respectively) and those of the Early Cretaceous in Asia (candidate trackmakers of *D. curriei*^6^).

Individual variation among samples of *Deltapodus* tracks has been described in other areas of the Iberian Peninsula, such as the Late Jurassic strata of Portugal^10^. The authors distinguished different morphotypes and proposed a possible ontogenetic influence in the samples with tracks with similar shapes but different sizes. Our studied sample is mainly composed of three size classes (small, medium and large individuals, Fig. S8), and some of the largest specimens show a higher FL/FW ratio and more elongated pes prints than those of smaller footprints. Thus, a possible ontogenetic explanation would be that larger specimens would have developed a larger heel pad than smaller individuals. However, this hypothesis does not apply to some of the specimens in CT-1, which are among the largest of the samples but show proportions similar to those of specimens belonging to the small size class (Fig. S10). In addition, the same individual produced tracks with high variation, as seen in the holotype trackway 1CA (Fig. 2) in CT-1 or in T1 in AG-3 (Fig. 3); therefore, the length of the footprints is dependent mainly on the impression of the heel pad and has a locomotory explanation in relation to the touch-down and weight-bearing phases during the pes–substrate interaction. The case of 1CA is possibly related to the variations in the state of the substrate (dryer or wetter) considering the differences previously reported in the CT-1 tracksite^18^ (Fig. S1). Thus, several of the analyzed tracks located in the wetter area are characterized by lower morphological preservation with a poorly preserved manus (mainly crescentic or semicircular in shape) and rather elongated pes with considerable rims that occasionally deform the manus. Size variation in individual tracks could also be explained by preservation, with larger tracks being undertracks^39^, so their proportions would be greater. Although this possibility exists and could explain the variations observed between different tracksites, it is not possible to explain the variations within trackway 1CA based only on undertrack phenomena.

**Figure 7:**
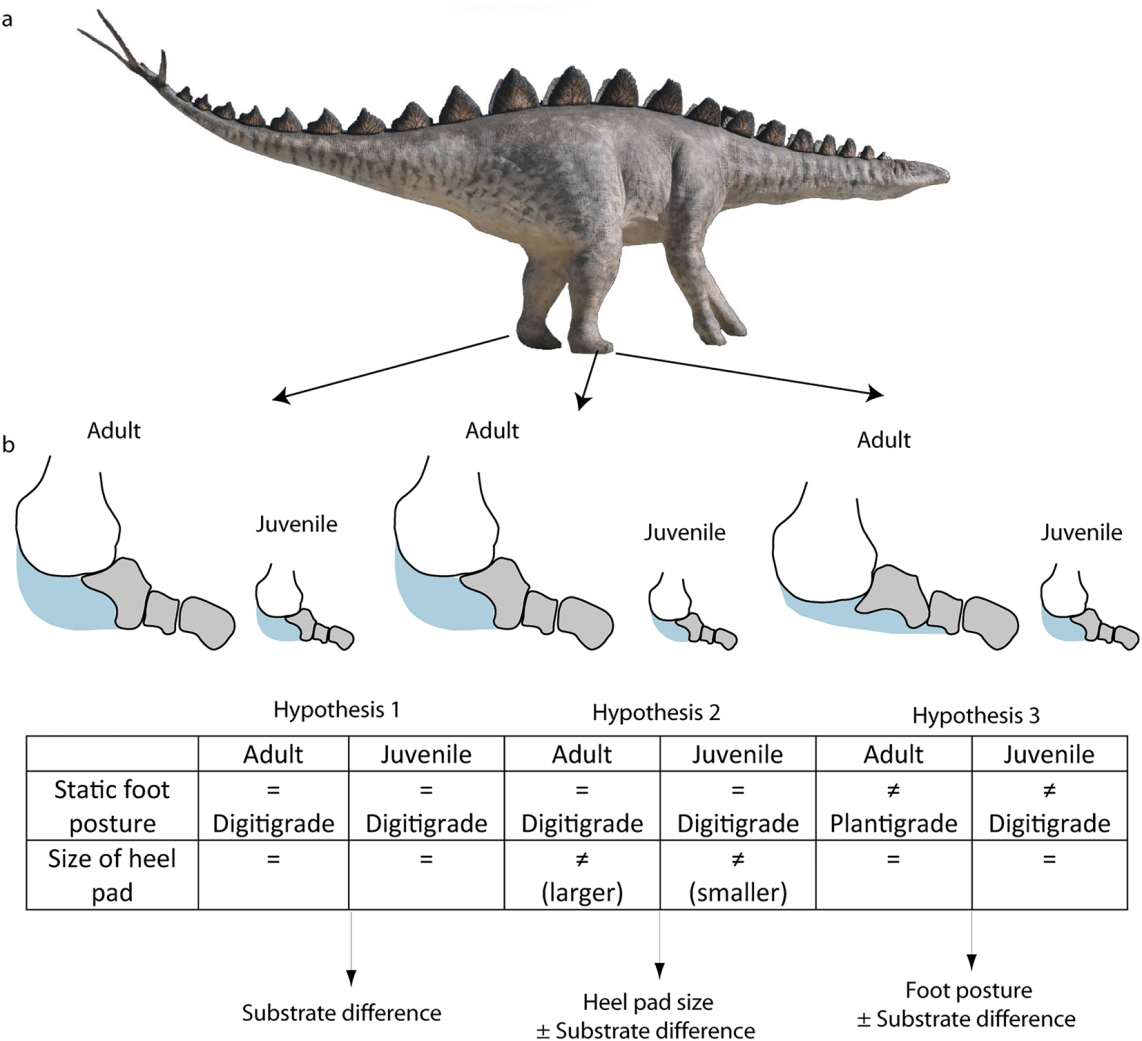
Proposed hypothesis (see^10,15,41^ and explanations in the text) for the interpretation of the causes of the variation in the size of the heel pad impression, assuming the same trackmaker species. (**a**) Reconstruction of *Dacentrurus*. (**b**) Model of the pes skeleton (based on *Kentrosaurus*^5^) to explain differences in the heel pad impressions. Note that the three hypotheses did not consider possible variations due to different species or sexual dimorphism.

Another ontogenetic explanation was proposed by Xing et al.^15^, who reported variations in the FL/FW ratio between individuals of different sizes, suggesting possible variations in foot posture (Fig. 7) between juveniles (digitigrade or subdigitigrade) and adults (plantigrade locomotion), considering shorter, tapered heel impressions in smaller footprints. Regarding this hypothesis, dinosaurs have been considered either digitigrades or subunguligrades, depending on the group (see^40,41^ and references therein). The latter authors^41^ have studied the development of soft tissue pads in sauropods, proposing a functionally plantigrade foot but retaining a “plesiomorphic skeletally digitigrade saurischian condition”. Among ornithischians, ornithopods are a “well-documented example of the transition from digitigrady to subunguligrady” during their evolution^40^. In both cases, the authors highlighted the importance of the soft tissue pad to reduce bone stresses, as well as in the evolution toward increases in body size and gigantism in the group^40,41^.

Among stegosaurs, little is known about whether the foot posture is more digitigrade or subunguligrade, considering the few osteological studies (including morpho-functional and/or biomechanical studies) on the available complete pes^3,5,38^ skeletons, but certainly, the trackmaker of *D. ibericus* is considerable in size considering the dimensions of the footprints. Cobos et al.^5^ proposed that the trackmaker would be semiplantigrade, showing an expanded impression in the metatarsal region that would represent a long heel reflection of the adipose tissue laid under the ankle that is reminiscent of that of extant elephants. Herrero-Gascón and Pérez-Lorente^20^ hypothesized a subdigitigrade pes with inclined metatarsals and low mobility among the phalanges, so the foot represents a rigid element. Guillaume et al.^10^ considered a digitigrade static posture but a plantiportal dynamic posture^41^. Considering the previous discussion and the available data, reconstructing the static foot posture based only on the footprint record is complicated, but it would be plausible to consider that the trackmaker of *D. ibericus* developed a considerable heel pad used in a plantiportal (functionally plantigrade) dynamic posture during locomotion^42^. Thus, the differences observed among the studied sample might have different explanations (Fig. 7) regarding the static foot posture and the size of the heel pad, which might be influenced by ontogenetic factors (possible differences in both during growth), and these factors were influenced by the state of the substrate. Thus, if larger and smaller individuals did not change the static foot posture or the size of the heel pad during their growth, the main differences would be related only to the substrate (Hypothesis 1). Nonetheless, larger individuals could have developed a proportionally larger heel pad than smaller individuals due to their greater weight (Hypothesis 2), or they could have acquired more plantiportal (functional plantigrade) locomotion (Hypothesis 3), as suggested by Xing et al.^15^. It should be noted that, for instance, in elephants, the bones in both the forefoot and hindfoot become slenderer and more robust, respectively, during growth, and they show ontogenetic changes in foot dynamics/spatiotemporal mechanics across the ontogeny^43^. Thus, we consider that for the studied sample the latter two hypotheses could be complementary based on the differences and the high variation in the FL/FW ratio related to the size classes and the partial overlap of the values but also because it is complicated to quantify and discard the complete influence of the substrate on the dimensions of the heel pad (Fig. 7).

In addition, considering the similarities in the metatarsals of several stegosaur species ^5,27,38^ and the fact that two main clades of stegosaurs inhabited the Iberian Peninsula during the Late Jurassic^5,25–28^, it is not possible to infer that some of the differences in the samples could also be a consequence of footprints produced by trackmakers of different species. Another possible biological explanation could even be sexual dimorphism, as suggested by some osteological features in different stegosaur species (see^5,44^ and references therein). Thus, further work is needed to understand whether different species and males/females could have developed different-sized heel pads and to determine their relationships with differences in body size and growth.

### Stegosaur versus sauropod tracks

Since the original description of *Deltapodus*, doubts about the interpretation of the trackmaker were raised, even when it was considered a “probable sauropod” in origin^3,4^. Similarly, since the first publications in the Iberian Peninsula, some of the tracks have been considered sauropod in origin (see ^5,18,22,24,29^ and references therein). These interpretations show the complexity of identifying stegosaur tracks. A comparison with sauropod footprints is necessary to establish criteria to distinguish between the two quadrupedal groups of dinosaurs that although show similarities in their tracks are phylogenetically distant from each other.

The Kimmeridgian-Berriasian interval in Europe has yielded copious amounts of data regarding quadrupedal dinosaur tracks produced by thyreophors and sauropods. Aside from *Deltapodus*, *Metatetrapous valdensis* from late Berriasian strata of northwestern Germany is the only thyreophor identified ichnotaxa and is attributed to ankylosaurs. The main differences between *Deltapodus* and *Metatetrapous* tracks are in the tetradactyl pes and manus, the latter is the most diagnostic feature of *Metatetrapous* compared with other ankylosaurian ichnotaxa. Interestingly, the debate between the candidate trackmaker as a thyreophor or a sauropod trackmaker has not occurred in this ichnotaxon, and their attribution to the former was based on the bearing of weak claws^45^.

**Figure 8:**
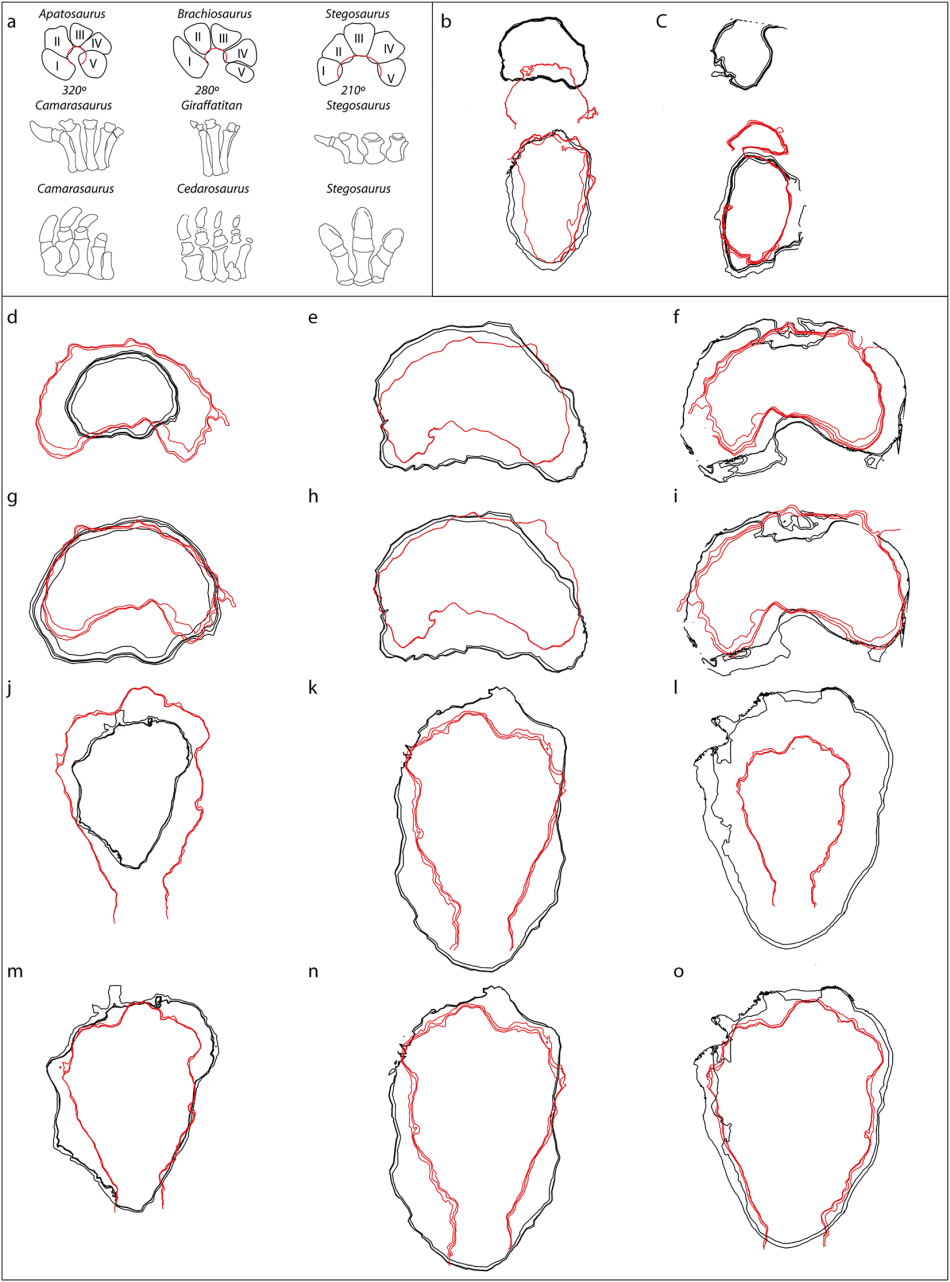
Differences between stegosaurs and sauropods. (**a**) Comparison of osteological manus and pes. Proximal view of the metacarpus of *Apatosaurus*, *Brachiosaurus* and *Stegosaurus* (redrawn from^46,50^; see references therein). Anterior view (oriented in the direction of travel of the animal) of the manus of *Camarasaurus*, *Giraffatitan* and *Stegosaurus* (redrawn from^2,51^; see references therein). Dorsal view of the pes of *Camarasaurus*, *Cedarosaurus* and *Stegosaurus* (redrawn from^2,51^; see references therein). (**b**) Comparison of a large *Deltapodus ibericus* (manus–pes set 1CA23) with the sauropod ichnotaxon *Iniestapodus burgensis*^51^ (manus–pes set LS7B3). (**c**) Comparison of small *Deltapodus ibericus* (manus–pes set AG3-4) with small sauropod tracks from Las Cerradicas^48^ (manus–pes set LCR13.6). Note the differences in manus and pes proportions and the manus–pes distance. (**d-i**) Manus of *D. ibericus* compared with a selection of sauropod footprints from the Las Cerradicas^48^, Las Sereas^51^ and Las Cuestas I^55^ tracksites at their real sizes (**d-f**) and manually scaled to similar sizes (**g-i**). (**d-g**) 1CA3m vs. LCR13.5m; (**e–h**) 1CA3m vs. LS7B3m; (**f-i**) 1CA3m vs. LCU-I-37-24m. **j-o**) Pes of *D. ibericus* compared with a selection of sauropod footprints from the Las Cerradicas^48^, Las Sereas^51^ and Las Cuestas I^55^ tracksites at their real size (**j-l**) and manually scaled to a similar size (**m–o**). (**j-m**) 1CA17p vs. LCR14.6p (MPZ2012-898). (**k-n**) 1CA17p vs. LS7B3p. (**l-o**) 1CA17p vs. LCU-I-37-12p. Note that *Deltapodus* outlines are drawn in red, and sauropod outlines are drawn in black. The outlines were generated with the software DigTrace  (https://www.digtrace.co.uk/).

In contrast, the debate about the trackmaker has concerned *Deltapodus* tracks in several areas. Similarities between stegosaur and sauropod manus prints have already been noted^5,22^. This similarity also has an osteological explanation since the metacarpal configuration is similar in both groups of dinosaurs^46^. The Kimmeridgian-Berriasian interval of the Iberian Peninsula has yielded well-preserved sauropod manus tracks that are also mainly kidney-shaped^47^. The manus proportions (FL/FW ratios) of those specimens range from 0.58 to 0.76^47^. These values partially overlap with those of our *D. ibericus* sample (0.27–0.71). García-Ramos et al.^22^ noted that stegosaur manus prints apparently had a lower FL and greater FW than sauropod manus prints, with FL/FW ratios lower than 0.5. This is the case in almost half of our studied specimens, and most of the sauropod tracks (n = 13) have values higher than 0.65 (only two *D. ibericus* specimens have higher values). This means that proportionally, sauropod manus are longer and more robust, and generally, stegosaur manus prints show slightly less antero-posterior development and are proportionally wider. This is clearly observed when the outlines of the manus prints of both groups are superimposed (Fig. 8) and compared with those of the main sauropod ichnotaxa (see Fig. 9 in^48^ and Fig. 6 in^49^ and references therein). This difference provides an osteological explanation because although both sauropods and stegosaurs have a semicircular vertical metacarpal configuration, the metacarpal arc is greater in coeval sauropods (e.g., *Brachiosaurus* and *Apatosaurus*) than in stegosaurs (e.g., *Stegosaurus*)^46,50^, with metacarpals I and V located in a more posterior position (Fig. 8a).

One of the reasons why both sauropod and stegosaur trackmakers could be confused when studying *Deltapodus* tracks might be that the pes prints are considerably elongated compared with other thyreophor tracks, including other stegosaur ichnotaxa [see Fig. 7 in^6^.and Fig. 9 in^8^]. Regarding the pes impressions of the Iberian Peninsula, Castanera et al.^47^ compiled information on 9 well-preserved specimens and reported that the FL/FW ratios ranged from 1.06 to 1.5. This means that sauropod tracks show wider medio-lateral development than *D. ibericus* tracks, whose footprints are proportionally longer and more elongated (1.35–1.92). A comparison of the outlines also clearly illustrates this difference (Fig. 8). Recently, Torcida et al.^51^ described a new sauropod ichnotaxon (*Iniestapodus*) from the Berriasian of the Iberian Peninsula, with striking similarities to those of *D. ibericus*. The FL/FW ratios (n = 6 trackways) of this ichnotaxon are 0.52–0.90 for the manus and 1.34–1.65 for the pes. These proportions partially overlap those of *D. ibericus* but the pes tracks are also proportionally wider and the the manus proportionally longer (Fig. 8). This is also supported by data from other European tracksites, with sauropod tracks from this temporal interval, such as *Parabrontopodus barkhausensis*^52^ (0.5–0.87/1.21–1.55) and *Rotundichnus*^53^ (pes values of 1.25–1.45) from Germany; *Parabrontopodus*-like tracks from Switzerland^54^ (0.5–0.83/1.18–1.58) and Spain^55^ (0.67/1.33); *Brontopodus plagnensis*^56^ (0.68/1.3) from France; and *Brontopodus*-like tracks from Spain^48^ (0.63–0.78/1–1.21). Compared with the main sauropod ichnotaxa (see Fig. 9 in^48^ and Fig. 6 in^49^ and references therein), the pes also seems to be wider and shorter than those of the *Deltapodus* tracks, in addition to the major differences in the presence of sharp claw impressions (generally long, curved and laterally deflected).

The differences in proportions in the pes also have an osteological explanation because the sauropod pes have five digits and metatarsals (see ^51^ and references therein), instead of the three present in stegosaurs^4,5,38^, which certainly would give a wider pes print (Fig. 8). This interpretation would mean that stegosaurs (at least the trackmakers of *Deltapodus ibericus*) developed a proportionally larger heel pad. Torcida et al.^51^ noted that the main differences between their studied sauropod tracks (*Iniestapodus*) and thyreophor tracks are the number, morphology and orientation of the claw marks, emphasizing blunt vs. sharp claw impressions. Thus, this would be the main characteristic for distinguishing between thyreophor and sauropod tracks, as observed in the osteological (Fig. 8a) and ichnological records. As shown here, the proportions of both manus and pes can also explain their origin, especially when sauropod tracks are within the size range of that of *Deltapodus* tracks (FL < 55 cm). In addition, Cobos et al.^5^ noted the short manus–pes distance in the *D. ibericus* trackway because of the short trunk in stegosaur trackmakers, which is also clearly illustrated when a manus–pes set of both groups is compared (Fig. 8b); therefore, although this parameter is clearly influenced by gait pattern, walking speed and ethological factors in quadrupedal dinosaurs^55,57^, this shorter manus–pes distance can also help in identifying stegosaur tracks.

### Notes on Stegosaur paleoecology: evidence of gregarious behavior

Gregarious behavior has been inferred from both the osteological and ichnological data of both saurischian and ornithischian dinosaurs. Among the former, this behavior in sauropods is the most documented^30–32,48^, whereas ornithopod dinosaurs are mainly the only group of ornithischians in which this kind of behavior has been described (see^30^ and references therein). Usually, sets of parallel trackways that show other features, such as similar preservation, speed values, close intertrackway spaces or pace rhythms, have been used to infer gregarious behavior among dinosaurs^30–32,48,58^, although the paleoenvironment also has a considerable influence in certain cases on trackway orientation^58–61^. To our knowledge, among stegosaurs, the only possible gregarious behavior reported in the literature comes from AG-3 and CT-1, which are some of the few tracksites where *Deltapodus* trackways have been described worldwide. In the former, Mampel et al.^16^ noted that trackways have the same orientation and proposed the hypothesis that trackmakers might show this kind of behavior. In CT-1, Alcalá et al.^18^ noted a dominant orientation of the footprints. The authors noted that “is difficult to discern any trackway”, emphasizing the observed dinoturbation processes would be produced by social groups (at least one group) composed different sized individuals, probably of different ages”.

The evidence observed in AG-3 (Fig. 3; Table S3) indicates two clear trackways showing complete parallelism between them heading to the NE and two other lineations (not three consecutive footprints to define a trackway) that are also heading in the same direction and are subparallel. The footprints show other features, such as similar preservation, close intertrackway spaces (especially between trackways 1 and 2 of less than a meter) and similar speed values (approximately 3 km/h), which support the hypothesis of Mampel et al.^16^. In CT-1, analysis of the cartography of the tracksite combined with 3D models of certain areas allowed us to distinguish at least 10 trackways or lineations (Figs. S1–S4), 7 of which follow a trajectory to the east. Other parameters are difficult to measure due to the high level of trampling of the surface. The lack of a bimodal orientation pattern in the trackways (including the theropod trackway) might indicate an absence of paleogeographical influence (see^61^ and references therein). García-Ortiz and Pérez-Lorente^30^ noted that footprint accumulations may also represent evidence of gregarious behavior; thus, although we cannot guarantee a gregarious behavior *sensu* *stricto* in the CT-1 tracksite, it might well be an example of such behavior *sensu* *lato* that although stegosaurs were not (or at least not all of them) strictly walking together at the same time, they inhabited these tidal flat environments^18^. The combination of the evidence in both AG-3 and CT-1 is very significant since these two examples are the only ichnological evidence described thus far in the fossil record that indicates that stegosaurs did exhibit gregarious behavior. In addition, this behavior is represented in two different ways (parallel trackways and accumulations showing preferred orientation) and in two different size classes (small and medium to large individuals); thus, it could also represent age segregation among stegosaur herds, as seen in sauropod dinosaurs^31^. Considering the new data in the studied sample, the absence of this kind of behavior in other areas might be biased not only by the scarcity of stegosaur trackways but also by possible misidentification of stegosaur tracks. Thus, the Teruel tracksites provide new information for tracking stegosaur tracks and their gregarious behavior.

## Material and methods

### Material

A review of the previously described and new occurrences of *Deltapodus* tracks from the Maestrazgo Basin has been carried out. Detailed descriptions of each tracksite are provided in the supplemental information. The size of the sample (n = 86; 37 individual pes tracks and 49 individual manus tracks) allowed us to analyze the variation among stegosaur tracks in a concrete area preserved in different paleoenvironments (Table S1) with various lithologies (limestones to sandstones) and preservation modes (either epireliefs or hyporeliefs). This is significant for determining whether the observed variability can be derived from preservation, locomotion or anatomical/biological factors (tracks produced by the same or different species or due to ontogenetic variations or any other biological cause). The two largest analyzed subsamples are located at the El Castellar (CT-1) and Aguilar 3 (AG-3, also known as El Rompido) tracksites. At the CT-1 tracksite, a review of the holotype trackway (1CA) of *D. ibericus* was carried out to provide new data on the morphological variations (20 tracks included) of the footprints along the type trackway. Nine manus–pes sets (some manus are missing) have been documented (see Table S1). In addition, 20 manus and 5 pes tracks from the previously published cartography^18^ of the tracksite were also individually analyzed. A northern area of the tracksite was still undescribed, and 7 manus and 5 pes were included in the sampled area. A cartography of this area of the tracksite is provided in the supplemental information (Fig. S4). In AG-3, a total of 16 tracks were analyzed. They include 6 manus–pes sets, some of which are part of the same trackway (see Fig. 3 and Table S3). The other studied materials are isolated manus, pes or manus–pes sets from tracksites CT-32, CT-64, LPV-1, FA-11, BDA, and AB-1. Details of the provenance of all the studied materials are provided in Table S1. Throughout the text, we have used the term Late Jurassic because many of the specimens are in Tithonian sediments, although the age of some specimens could be early Berriasian (Table S1). The manus and pes were analyzed individually. The acronyms used for each track represent the name of the tracksite and a correlative number within the site. Recovered specimens (e.g., MAP-8430; MAP-8446) are housed at the Museo Aragonés de Paleontología and are labeled with the acronym MAP and the correlative number in the collection.

### 3D modeling and data extraction

The Aguilar 3 tracksite was scanned with two different instruments, i.e., a structured-light 3D scanner (Artec mod. MHT) with a precision of 500 µm and a phase-shift 3D laser scanning system (Leica HDS6100). In addition, all the material (every individual track) was photogrammetrically documented. Three-dimensional photogrammetric models were built using the Agisoft Photoscan Professional Edition software, with approximately 10–20 photographs per track. Pictures were taken with a Pentax K200D camera. The photogrammetric meshes used in this study are available for download in the supplementary information (10.6084/m9.figshare.26028919), following the recommendation of Falkingham et al.^62^. Posteriorly, the 3D models, exported as OBJ files, were processed in CloudCompare (v.2.13. alpha) to obtain false-color depth maps using the color schemes provided by Belvedere^63^. Individual parameters of each footprint were obtained with ImageJ software. Several morphometric parameters were measured to characterize and compare the ichnites (see Fig. S11, Table S2). These parameters are the Footprint Length (FL), Footprint Width (FW), Digit II, III and IV lengths (DII, DIII, DIV, respectively), Anterior Width (AW), and Posterior Width (PW). FLdef represents the footprint length considering the deformation of the rims. The II-III and III-IV divarication angles and the manus–pes distance (D-mp) were also measured. Dm-p1 represents the manus–pes distance measured from the middle of the tracks, and Dm-p2 represents the distance measured from the posterior part of the manus to the anterior part of the pes. The heteropody index was calculated following the formula proposed by Gonzalez Riga^64^ (FL manus × FW manus/FL pes × FW pes). The FL/FW ratio was estimated to quantify the dimensions of the tracks. Bivariate graphs to visualize the data were constructed using PAST software^65^. The morphological preservation (MP) of each track was evaluated according to the scale of Marchetti et al.^66^. Different size classes were distinguished based on the range of sizes of *Deltapodus* tracks described in the literature (e.g., ^7, 8, 10,15^ and references therein) on the basis of the pes footprint length (FL) as follows: (1) tiny = FL < 15 cm; (2) small = 15 cm < FL < 30 cm; (3) medium = 30 cm < FL < 45 cm; and (4) large = FL > 45 cm. Individual tracks were computationally compared with the DigTrace^67^ v.1.8.1 software. Stat tracks (e.g., the mediotype *sensu* Belvedere et al.^68^) were constructed using 4 pes impressions (1CA3p, 1CA17p, 1CA23p, and 1CA27p) and 2 manus (1CA3m and 1CA23m) for the holotype of *D. ibericus*. The mediotype was constructed with a selection of 8 landmarks in the pes and 4 in the manus (Fig. S11). A comparison of the outlines between the sample and a selection of sauropod tracks was carried out. The sauropod tracks come from different tracksites located in Spain and belong to different ichnotaxa; these include *Brontopodus*-like tracks from Las Cerradicas^48^ (manus LCR13.5m and manus–pes set LCR13.6 are located at the tracksite, and pes MPZ2012-898 is housed at the Museo de Ciencias Naturales de la Universidad de Zaragoza and corresponds with track LCR14.6p at the tracksite); *Iniestapodus burgensis* (manus–pes set LS7B3) from Las Sereas^51^ and *Parabrontopodus*-like tracks from Las Cuestas I^55^ tracksites (manus LCU-I-37-24m and pes LCU-I-37-12p, which are housed at the Museo Numantino de Soria and have the acronyms MNS2006/75/3 and MNS2006/75/1, respectively).

Locomotion speed was estimated using the Alexander formula^69^, i.e., speed (v) = 0.25 g^0.5^*SL^1.67^*h*^−1.17^, where g = 9.8 and is the acceleration due to gravity; SL = stride length; and h = the hip height that is estimated with the formula h = 6FW, according to Cobos et al.^5^. The aim of analyzing these data is to compare the relative speed values among the trackways rather than the absolute speed values.

### Supplementary Information

Supplementary Information 1.

Supplementary Table 1.

Supplementary Table 2.

Supplementary Table 3.

## Data Availability

The datasets generated during the current study are available in the supplementary information. 3D models of the dinosaur footprints are also available for download in Figshare (10.6084/m9.figshare.26028919).

## References

[1] Maidment SC (2010). Stegosauria: A historical review of the body fossil record and phylogenetic relationships. Swiss J. Geosci..

[2] Thulborn T (1990). Dinosaur tracks.

[3] Whyte MA, Romano M (1994). Probable sauropod footprints from the Middle Jurassic of Yorkshire, England. Gaia.

[4] Whyte MA, Romano M (2001). Probable stegosaurian dinosaur tracks from the Saltwick Formation (Middle Jurassic) of Yorkshire England. Proc. Geol. Assoc..

[5] Cobos A, Royo-Torres R, Luque L, Alcalá L, Mampel L (2010). An Iberian stegosaurs paradise: The Villar del Arzobispo Formation (Tithonian–Berriasian) in Teruel (Spain). Palaeogeogr. Palaeoclimatol. Palaeoecol..

[6] Xing L (2013). First record of
* Deltapodus
* tracks from the early cretaceous of China. Cretac. Res..

[7] Mateus O, Milàn J, Romano M, Whyte MA (2011). New finds of stegosaur tracks from the upper Jurassic Lourinhã Formation. Portugal. Acta Palaeontol Pol..

[8] Pascual-Arribas C, Hernández-Medrano N (2015). Nuevas huellas de estegosáuridos en el Titoniense-Berriasiense de la Cuenca de Cameros (Formación Magaña). Rev. Soc. Geol. Esp..

[9] dePolo PE (2020). Novel track morphotypes from new tracksites indicate increased Middle Jurassic dinosaur diversity on the Isle of Skye, Scotland. PLoS One.

[10] Guillaume ARD, Costa F, Mateus O (2022). Stegosaur tracks from the Upper Jurassic of Portugal: New occurrences and perspectives. Ciênc. da Terra..

[11] Milàn J, Chiappe LM (2009). First American record of the Jurassic ichnospecies *Deltapodus brodricki* and a review of the fossil record of stegosaurian footprints. J. Geol..

[12] Lockley MG (2017). New dinosaur track occurrences from the Upper Jurassic Salt Wash Member (Morrison Formation) of Southeastern Utah: Implications for thyreophoran trackmaker distribution and diversity. Palaeogeogr. Palaeoclimatol. Palaeoecol..

[13] Lockley MG, Foster JR, Hunt Foster R (2018). The first North American
* Deltapodus
* trackway in a diverse
* Anomoepus
*
, theropod, sauropod, and turtle track assemblage from the Upper Jurassic salt wash member (Morrison Formation) of eastern Utah. Bull. NM Mus. Nat. Hist. Sci..

[14] Belvedere M, Mietto P (2010). First evidence of stegosaurian *Deltapodus* footprints in North Africa (Iouaridène Formation, Upper Jurassic, Morocco). Palaeontology.

[15] Xing L (2021). Stegosaur track assemblage from Xinjiang, China, featuring the smallest known stegosaur record. Palaios.

[16] Mampel L (2010). Icnitas de dinosaurios en Aguilar del Alfambra (Teruel, España). Teruel.

[17] Alcalá L (2012). Icnitas de dinosaurios en la Formación Villar del Arzobispo de Ababuj (Teruel, España). Geogaceta.

[18] Alcalá L (2014). Preservation of dinosaur footprints in shallow intertidal deposits of the Jurassic-Cretaceous transition in the Iberian Range (Teruel, Spain). Ichnos.

[19] Campos-Soto S (2017). Jurassic Coastal Park: A great diversity of palaeoenvironments for the dinosaurs of the Villar del Arzobispo Formation (Teruel, eastern Spain). Palaeogeogr. Palaeoclimatol. Palaeoecol..

[20] Herrero Gascón J, Pérez-Lorente F (2017). Hoof-like unguals, skin, and foot movements deduced from *Deltapodus* casts of the Galve Basin (Upper Jurassic-Lower Cretaceous, Teruel, Spain). Ichnos.

[21] Pascual C, Canudo JI, Hernández N, Barco JL, Castanera D (2012). First record of stegosaur dinosaur tracks in the Lower Cretaceous (Berriasian) of Europe (Oncala group, Soria, Spain). Geodiversitas.

[22] García-Ramos, J. C., Piñuela L., Ruiz-Omeñaca, J. I. Pereda Suberbiola, X. *Costas jurásicas frecuentadas por estegosaurios*. In Libro de resúmenes. XXIV Jornadas de la Sociedad Española de Paleontología (eds. Ruiz-Omeñaca, J.I., Piñuela, L & García-Ramos, J. C) 33–34 (Museo del Jurásico de Asturias, Colunga, 2008).

[23] Lockley MG, García-Ramos JC, Piñuela L, Avanzini M (2008). A review of vertebrate track assemblages from the Late Jurassic of Asturias, Spain with comparative notes on coeval ichnofaunas from the western USA: Implications for faunal diversity in siliciclastic facies assemblages. Oryctos.

[24] Piñuela, L., García-Ramos, J. C., Fernández, L. A. & Ruiz-Omeñaca, J. I. *Nuevos rastros de estegosaurios en el Jurásico Superior de Asturias*. In XXX Jornadas de Paleontología de la Sociedad Española de Paleontología. ¡Fundamental! (eds. Royo-Torres, R., Verdú, F. J. & Alcalá, L.), **24**, 187–190 (2014).

[25] Escaso F (2007). New evidence of shared dinosaur across Upper Jurassic proto-North Atlantic: *Stegosaurus* from Portugal. Naturwissenschaften.

[26] Mateus O, Maidment SC, Christiansen NA (2009). A new long-necked ‘sauropod-mimic’stegosaur and the evolution of the plated dinosaurs. Proc. R. Soc. Lond. B.

[27] Costa F, Mateus O (2019). Dacentrurine stegosaurs (Dinosauria): A new specimen of *Miragaia longicollum* from the Late Jurassic of Portugal resolves taxonomical validity and shows the occurrence of the clade in North America. PLoS One.

[28] Sánchez-Fenollosa S, Suñer M, Cobos A (2022). New fossils of stegosaurs from the Upper Jurassic of the Eastern Iberian Peninsula (Spain). Diversity.

[29] Gascón JH, Pérez Lorente F (2012). El Rompido (Aguilar del Alfambra). Icnitas de dinosaurios en la Formación Villar del Arzobispo. Teruel. Geogaceta.

[30] García-Ortiz E, Pérez-Lorente F (2014). Palaeoecological inferences about dinosaur gregarious behaviour based on the study of tracksites from La Rioja area in the Cameros Basin (Lower Cretaceous, Spain). J. Iber. Geol..

[31] Myers TS, Fiorillo AR (2009). Evidence for gregarious behavior and age segregation in sauropod dinosaurs. Palaeogeogr. Palaeoclimatol. Palaeoecol..

[32] Castanera D (2014). Gregarious behaviour inferred from sauropod footprints in the Iberian Peninsula: Taxonomic, palaeoecological and palaeoenvironmental implications. J. Iber. Geol..

[33] Aurell M (2016). Stratigraphy and evolution of the Galve sub-basin (Spain) in the middle Tithonian–early Barremian: Implications for the setting and age of some dinosaur fossil sites. Cretac. Res..

[34] Campos-Soto S (2019). Revisiting the age and palaeoenvironments of the Upper Jurassic-lower cretaceous? Dinosaur-bearing sedimentary record of eastern Spain: Implications for Iberian palaeogeography. J. Iber. Geol..

[35] Aurell M (2019). Kimmeridgian-Berriasian stratigraphy and sedimentary evolution of the central Iberian Rift System (NE Spain). Cretac. Res..

[36] Bádenas B, Aurell M, Gasca JM (2018). Facies model of a mixed clastic–carbonate, wave-dominated open-coast tidal flat (Tithonian–Berriasian, north-east Spain). Sedimentology.

[37] Val J, Aurell M, Badenas B, Castanera D, Subias S (2019). Cyclic carbonate–siliciclastic sedimentation in a shallow marine to coastal environment (latest Kimmeridgian–early Tithonian, Galve sub-basin, Spain). J. Iber. Geol..

[38] Gierlinski G, Sabath K (2002). A probable stegosaurian track from the Late Jurassic of Poland. Acta Palaeontol. Pol..

[39] Milàn J, Bromley RG (2006). True tracks, undertracks and eroded tracks, experimental work with tetrapod tracks in laboratory and field. Palaeogeogr. Palaeoclimatol. Palaeoecol..

[40] Moreno K, Carrano MT, Snyder R (2007). Morphological changes in pedal phalanges through ornithopod dinosaur evolution: A biomechanical approach. J. Morphol..

[41] Jannel A, Salisbury SW, Panagiotopoulou O (2022). Softening the steps to gigantism in sauropod dinosaurs through the evolution of a pedal pad. Sci. Adv..

[42] Michilsens F, Aerts P, Van Damme R, D'Août K (2009). Scaling of plantar pressures in mammals. J. Zool..

[43] Panagiotopoulou O, Pataky TC, Hill Z, Hutchinson JR (2012). Statistical parametric mapping of the regional distribution and ontogenetic scaling of foot pressures during walking in Asian elephants (*Elephas maximus*). J. Exp. Biol..

[44] Barden HE, Maidment SC (2011). Evidence for sexual dimorphism in the stegosaurian dinosaur *Kentrosaurus aethiopicus* from the Upper Jurassic of Tanzania. J. Vertebr. Paleontol..

[45] Hornung JJ, Reich M (2014). *Metatetrapous valdensis* Nopcsa, 1923 and the presence of ankylosaur tracks (Dinosauria: Thyreophora) in the Berriasian (early cretaceous) of Northwestern Germany. Ichnos.

[46] Senter P (2010). Evidence for a sauropod-like metacarpal configuration in stegosaurian dinosaurs. Acta Palaeontol. Pol..

[47] Castanera D, Falkingham PL, Marty D, Richter A (2016). Iberian sauropod tracks through time: Variations in sauropod manus and pes track morphologies. Dinosaur tracks The next steps.

[48] Castanera D (2011). New evidence of a herd of titanosauriform sauropods from the lower Berriasian of the Iberian range (Spain). Palaeogeogr. Palaeoclimatol. Palaeoecol..

[49] Tomaselli MB (2022). New titanosaurian sauropod tracks with exceptionally well-preserved claw impressions from the Upper Cretaceous of Argentina. Cretac. Res..

[50] Senter P (2011). Evidence for a sauropod-like metacarpal configuration in ankylosaurian dinosaurs. Acta Palaeontol. Pol..

[51] Torcida Fernández-Baldor F, Díaz-Martínez I, Huerta P, Montero Huerta D, Castanera D (2021). Enigmatic tracks of solitary sauropods roaming an extensive lacustrine megatracksite in Iberia. Sci. Rep..

[52] Meyer CA, Belvedere M, Englich B, Lockley MG (2021). A reevaluation of the Late Jurassic dinosaur tracksite Barkhausen (Wiehengebirge, Northern Germany). Palaentolog Z.

[53] Lockley MG, Wright JL, Thies D (2004). Some observations on the dinosaur tracks at Münchehagen (Lower Cretaceous), Germany. Ichnos.

[54] Marty D (2010). Comparative analysis of late Jurassic sauropod trackways from the Jura Mountains (NW Switzerland) and the central High Atlas Mountains (Morocco): Implications for sauropod ichnotaxonomy. Hist. Biol..

[55] Castanera D, Pascual C, Canudo JI, Hernandez N, Barco JL (2012). Ethological variations in gauge in sauropod trackways from the Berriasian of Spain. Lethaia.

[56] Mazin JM, Hantzpergue P, Olivier N (2017). The dinosaur tracksite of Plagne (early Tithonian, Late Jurassic; Jura Mountains, France): The longest known sauropod trackway. Geobios.

[57] Gonzalez Riga BJ, Tomaselli MB (2019). Different trackway patterns in titanosaur sauropods: analysis of new Titanopodus tracks from the Upper Cretaceous of Mendoza, Neuquén Basin, Argentina. Cretac. Res..

[58] Ostrom JH (1972). Were some dinosaurs gregarious?. Palaeogeogr. Palaeoclimatol. Palaeoecol..

[59] Razzolini NL (2016). Ichnological evidence of megalosaurid dinosaurs crossing Middle Jurassic tidal flats. Sci. Rep..

[60] Getty PR (2017). Perennial lakes as an environmental control on theropod movement in the Jurassic of the Hartford Basin. Geosciences.

[61] Castanera D (2023). Paleoecology and paleoenvironment of the Early Cretaceous theropod-dominated ichnoassemblage of the Los Corrales del Pelejón tracksite, Teruel Province, Spain. Palaeogeogr. Palaeoclimatol. Palaeoecol..

[62] Falkingham PL (2018). A standard protocol for documenting modern and fossil ichnological data. Palaeontology.

[63] Belvedere, M. *CloudCompare Depth Map color schemes*, Figshare. Dataset. 10.6084/m9.figshare.11742660.v3. (2020)

[64] Gonzalez Riga BJ, Calvo JO (2009). A new wide-gauge sauropod track site from the Late Cretaceous of Mendoza, Neuquén Basin, Argentina. Palaeontol..

[65] Hammer Ø, Harper DA (2001). Past: Paleontological statistics software package for educaton and data anlysis. Palaeontol. Electron..

[66] Marchetti L (2019). Defining the morphological quality of fossil footprints. Problems and principles of preservation in tetrapod ichnology with examples from the Palaeozoic to the present. Earth-Sci. Rev..

[67] Bennett MR, Budka M, Bennett MR, Budka M (2019). Introduction to DigTrace. Digital technology for forensic footwear analysis and vertebrate ichnology.

[68] Belvedere M (2018). Stat-tracks and mediotypes: Powerful tools for modern ichnology based on 3D models. PeerJ.

[69] Alexander RM (1976). Estimates of speeds of dinosaurs. Nature.

