# Supplementary material for: The complexity of tracking stegosaurs and their gregarious behavior
Source: Sci Rep. 2024 Jul 3;14:14833. doi: 10.1038/s41598-024-64298-9 (PMC11222438; doi:10.1038/s41598-024-64298-9)
Supplement: Supplementary file 1 — Supplementary Information 1. [file 41598_2024_64298_MOESM1_ESM.pdf]

Description of the footprints in the different tracksites of the Maestrazgo Basin

CT-1/El Castellar tracksite:

This site is composed of two track-bearing levels 1CT-1 and 2CT-1. 2CT-1 is a large surface of more than 500 m^2 with several areas where more than 800 tracks have been estimated[S1]. The site is preserved in the mudstone and peloidal limestone facies of Campos-Soto et al.[S2]and the tracks are preserved as concave epireliefs. Alcalá et al.[S1] provided a cartography (Fig.S1) of an area of 230m^2. The identification of stegosaur tracks at this site is particularly complex because of the moderate to high dinoturbation index[S1,S3-S4]. In addition, manus dominate with respect to the pes prints so the identification of trackways is also a difficult task. A review of the site has been carried out to show: 1) the variations among the holotype trackway of *D. ibericus*; 2) to identify possible trackways; and 3) to analyze the better-preserved tracks (mainly manus prints) in the previously published area[S1] to determine whether they might belong to *D. ibericus* or to another ichnotaxa (including the possibility of a sauropod origin).

<u>Analysis of the *D. ibericus* holotype trackway:</u>

The holotype of *D. ibericus* is a 23 m long trackway (1CA) composed of 30 manus-pes sets (Fig.S1; Fig.2 in the main text), and only a few present the characteristic blunt-toed, elongate, subtriangular tridactyl pes track of *Deltapodus*. Among them, only a few manus and pes tracks (1CA3m, 1CA3p, 1CA17p, 1CA23m, 1CA23p, and 1CA27p) (see the mediotype built with these footprints in Fig.2c, 2c in the main text) exhibited a medium degree of morphological preservation, while the others have lower degree. The manus prints vary from different shapes, according to morphological preservation, from kidney-shaped (higher MP) to crescent or semicircular (lower MP). The best-preserved manus prints are kidney-shaped, with no clear evidence of pollex mark impressions and with a FL/FW ratio of 0.36-0.44 (in 1CA3m and 1CA23m). Crescent-shaped manus prints are generally influenced by the pes overstepping the manus (or partially modifying the posterior area). Manus prints with semicircular morphology are a consequence of the manus touching the substrate with the posteriormost part. The manus dimensions vary between 0.13-0.2 (FL) and 0.2-0.38 (FW), and the FL/FW ratio ranged from 0.36-0.58.

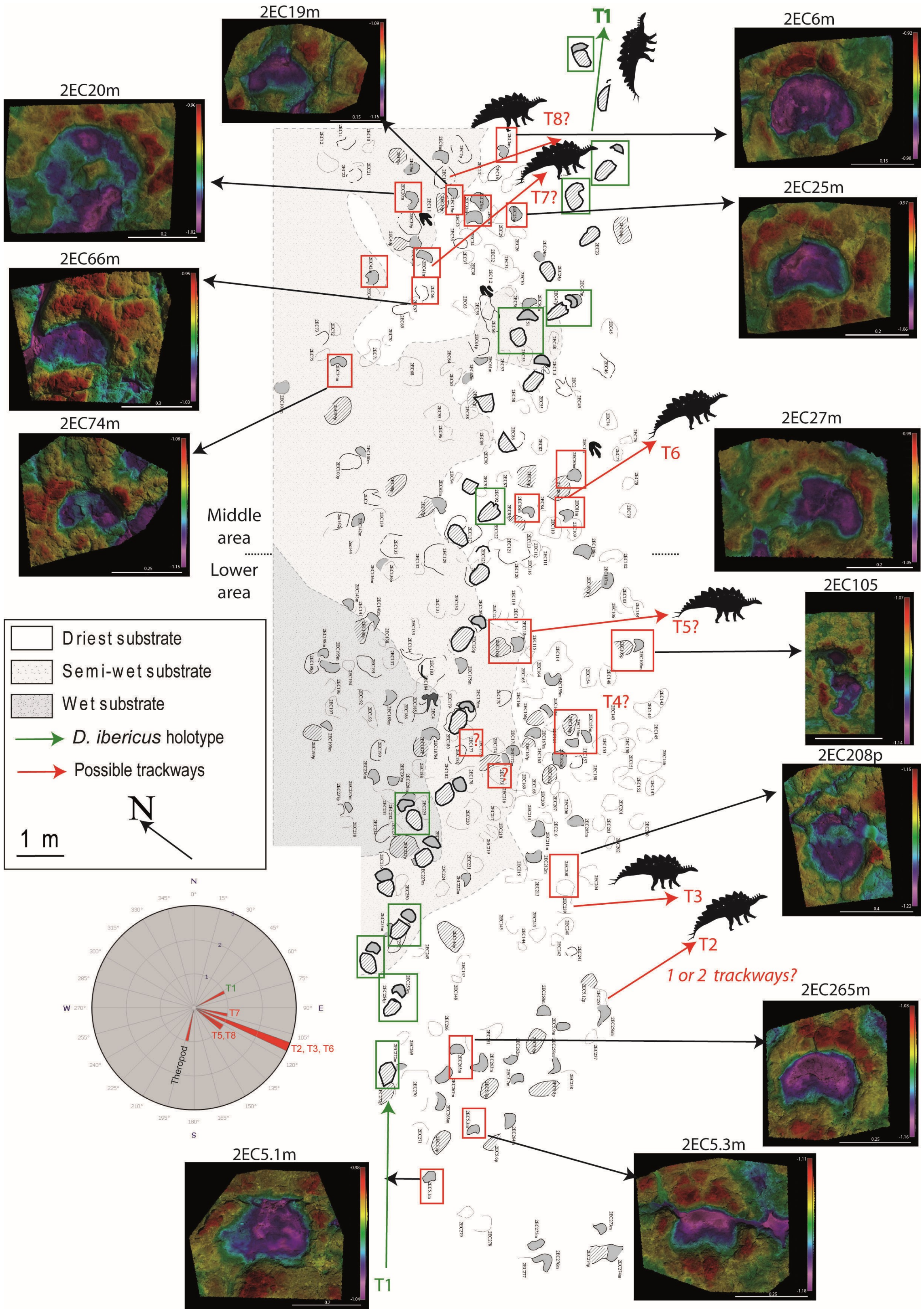

Fig. S1: Sketch map of the lower and middle areas of the main surface of the CT-1 tracksite (from[S1]) showing false-color depth maps of representative manus and pes tracks. Note the interpreted possible trackways or lineations. The rose digram indicates the orientation of the possible (mainly Deltapodus*) trackways.*

Pes prints also show morphological variations from subtriangular shape (reversed delta, showing high variation between anterior and posterior width) to subrectangular shape (low variations in the anterior and posterior width), with poorly preserved footprints being oval impressions. Cobos et al.[S3] reported a mean pes length of 0.44 m and a mean pes width of 0.3 for pes tracks, and the FL/FW ratio was 1.48. These data are consistent with those obtained here (see Table S2), where they vary between 0.4-0.54 m (FL), 0.21-0.35 m (FW) and 1.57-1.92 (FL/FW). It is noteworthy that the considerable variation in these data is a consequence of the variable preservation of the heel pad impressions, which makes some footprints more elongated than others. As a consequence of the variation in pes and manus morphology, heteropody also shows considerable variation through the trackway varying from medium (1:3.7) to low (1:2). Interestingly, track 1CA17p shows a subtriangular heel pad morphology but with a subrectangular outline (although the posterior part of the footprint is broken). A particular feature observed along the pes of the *Deltapodus ibericus* holotype trackway is in the impressions of the distal part (the hoof-like impression) of digit IV, which are generally poorly impressed and slightly shorter than those of digits II and III, as observed in tracks 1CA3p, 1CA17p, and 1CA23p.

The trackway features are characterized by a "moderately wide internal width (range 0.05–0.23 m)" that varies due to a slight change in the direction of travel (Cobos et al.[S3]). Manus prints are located in front of the pes, with a varied location from more laterally to medially, and in all cases, the manus-pes distance is short. Both manus and pes are characterized by considerable (approximately 35°) outward rotation. The estimated speed of the trackway is approximately 2.74 km/h, and the direction of travel is N20°W.

Analysis of other tracks in the CT-1 tracksite:

A review of the better-preserved manus and pes (Figs. S1-S3) tracks at the site provides interesting data on the variation in possible *Deltapodus* tracks. The tracksite has been divided into three areas (lower, middle and upper areas; see Figs. S1-S4), which are currently separated by wood walkways that allow tourists to visit the site. In the lower area, five different pes prints with consistent FL (0.41-0.47 m) and FW (0.25-0.33 m) values that fall close to the range of the holotype of *D. ibericus* have been analyzed. The footprints have the characteristic delta/subrectangular shape of this ichnospecies, and some specimens also show a better impression of digits II-III than digit IV, similar to the holotype. It should be highlighted that track EC208p (Figs. S1-S2) has a high MP value and the characteristic blunt-toed pes track of *Deltapodus*, but the track is not as elongated (FL/FW ratio = 1.4) as those observed in the holotype of *D. ibericus*, especially 1CA23p. The other pes impressions are more consistent in terms of the FL/FW ratio with those of the holotype. The analyzed manus prints in this area of the tracksite vary from kidney to semicircular in shape with rather consistent FL and FW values (0.13-0.2 and 0.23-0.33, respectively). Nonetheless, the manus prints had a slightly greater FL/FW ratio (0.52-0.62) than the holotype.

In the lower area of the site, trackways are difficult to identify due to the high dinoturbation index and the weathering of some tracks. The trackway 1CA, the holotype, starts in the lower part of this area, showing a NE orientation. In addition, other tracks are oriented, and at least 4/5 lineations (T2-T5) can be discerned (Fig.S1), all which are heading to the E.

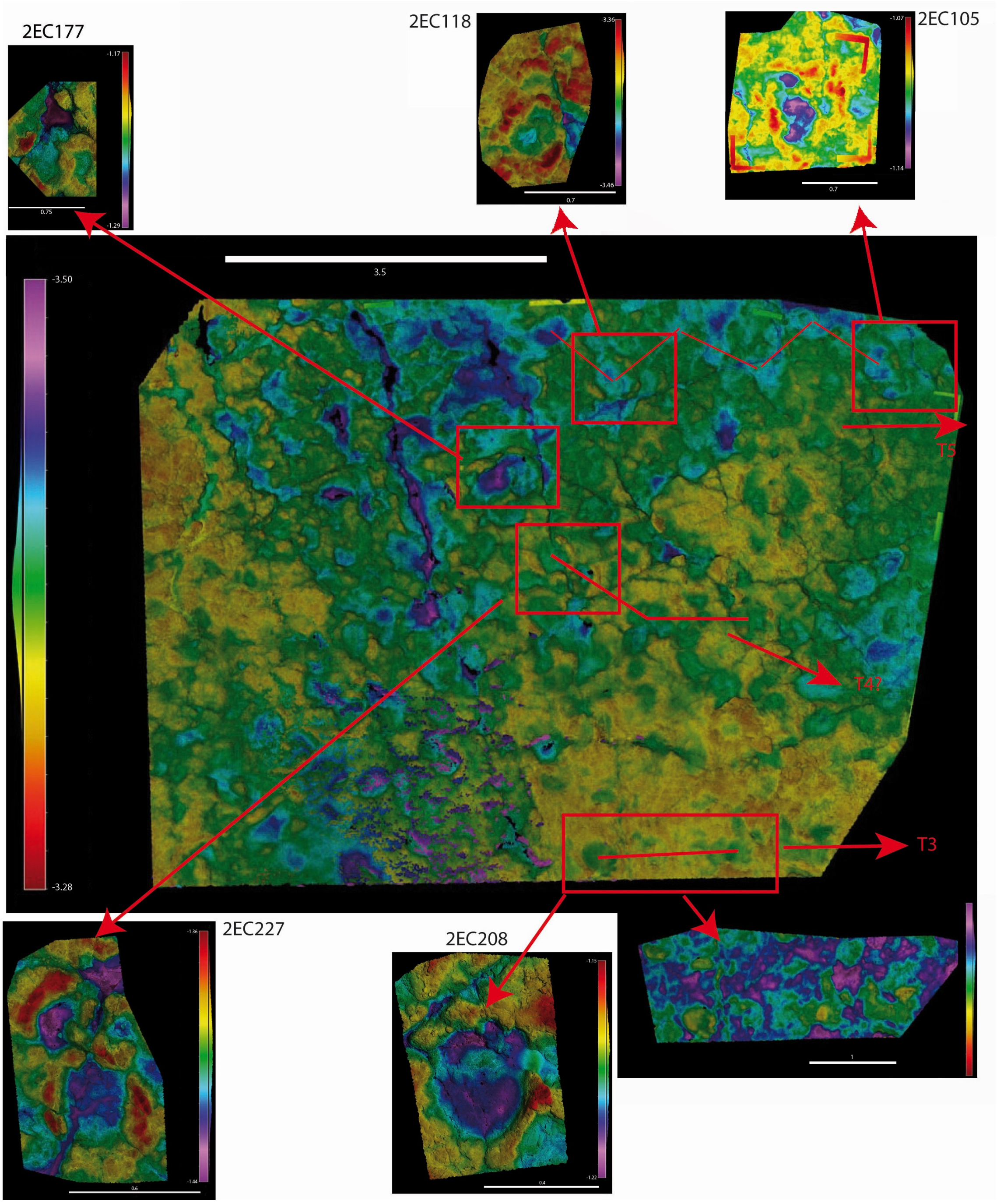

Fig. S2: False color depth map of part of the lower area of the CT-1 tracksite showing representative manus and pes tracks and possible lineations (trackways T3-T5 in Fig. S1). Note the difference in the preservation of the hoof-like impressions in the 2EC208 specimen.

The middle area (Fig. S1) is also characterized by a high dinoturbation index and a semiwet substrate (see Fig. S1 and Alcalá et al.[S1]), where several manus prints with

high morphological preservation can be identified. They possibly belong to three different manus-dominated trackways (T6-T8 in Fig.S1), heading subparallel to the E, and T7-T8 with a trajectory that crosses the holotype trackway. Manus morphology (Fig. S3) varies from kidney to semicircular, showing dimensions similar to those of the holotype (FL= 0.10-0.20, FW= 0.17-0.30), with proportions mainly within the range, but enlarging the upper limit (FL/FW = 0.41-0.66). It should be noted the variations observed between tracks that have similar FL values, but their width is considerably greater.

The unpublished upper area (Fig. S4) also shows a high dinoturbation index with possibly at least 2 different trackways, including manus and pes tracks. A total of 12 footprints, 7 from manus and 5 from pes, have been analyzed. The dimensions of the tracks are slightly lower than those of the holotype trackway on both the manus (FL=0.09-0.16; FW =0.2-0.36) and the pes (FL=0.37-0.56; FW = 0.25-0.36) tracks. The dimensions of the manus mainly fall within the range of the holotype, also slightly expanding the upper limit (FL/FW ratio = 0.42-0.64), whereas the pes (FL/FW ratio = 1.4-1.55) do not fall within the range of variation but are closer to the lower values. These data are more similar to the previously mentioned track EC208p.

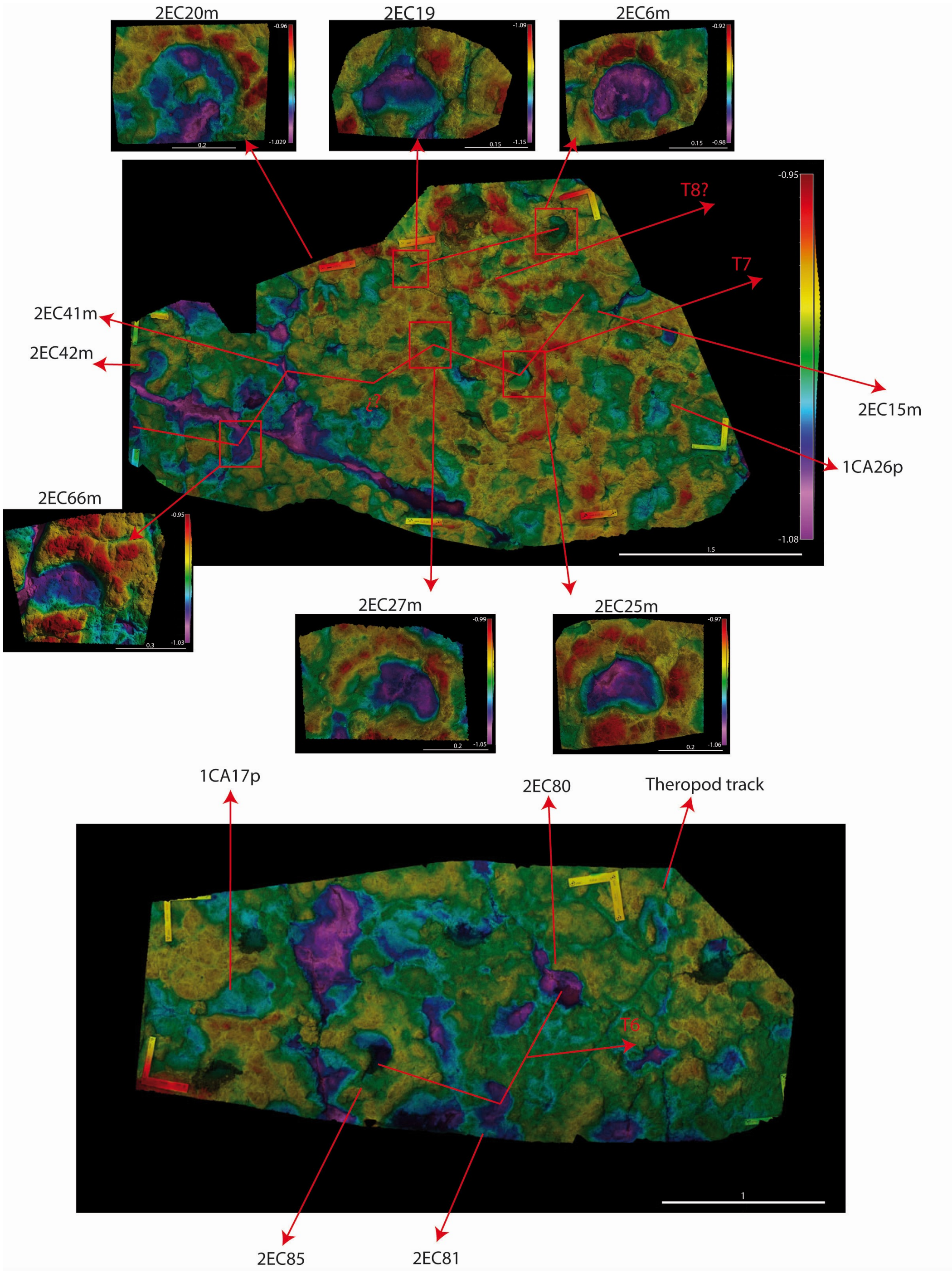

Fig. S3: False color depth map of two areas of the middle part of the CT-1 tracksite showing representative manus and pes tracks and three lineations corresponding to possible trackways T6-T8 (see Fig. S1).

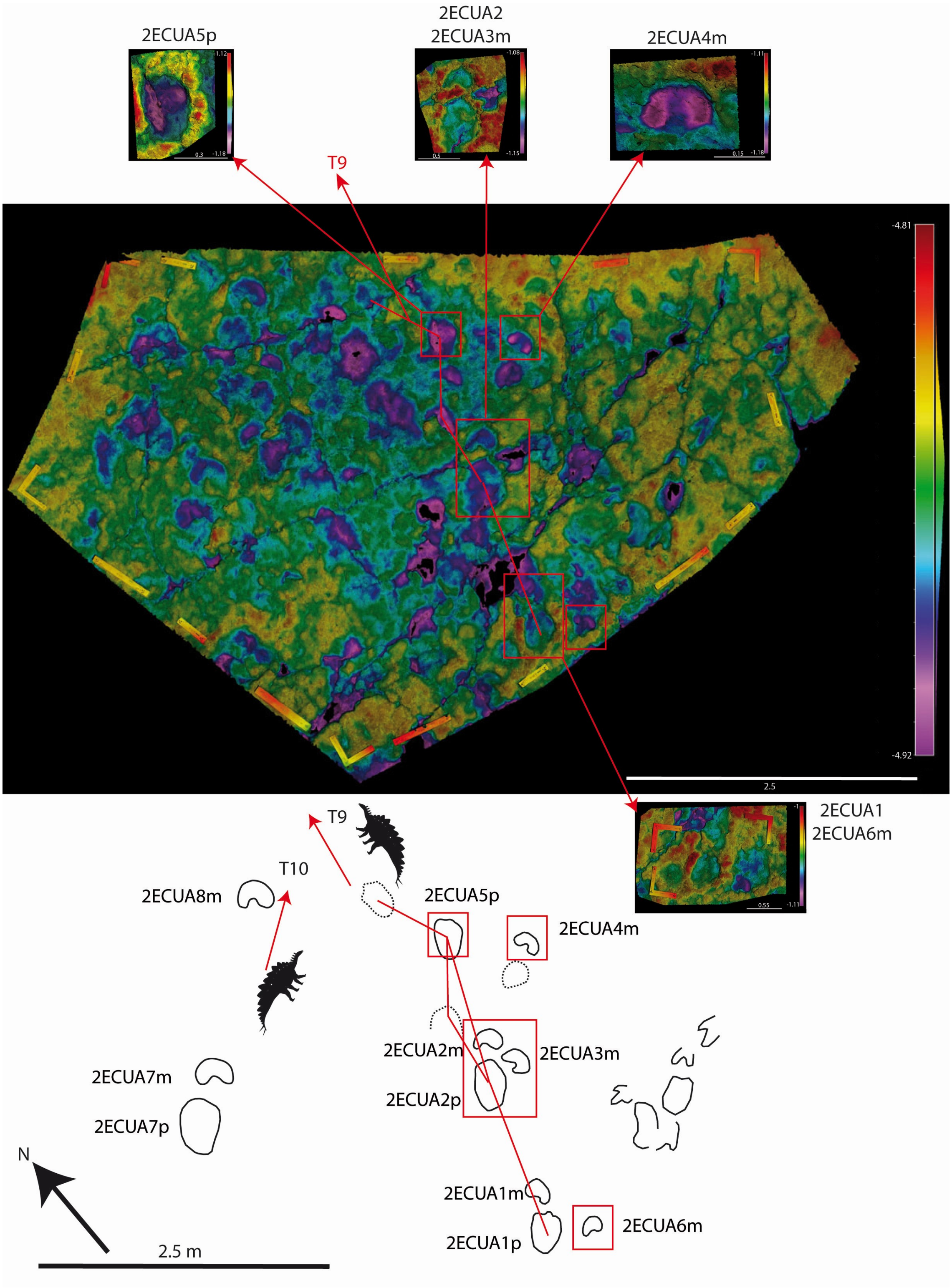

Fig. S4: A) False color-depth map of the upper area of the CT-1 tracksite. B) Sketch map of the upper area of the CT-1 tracksite. Note that only the tracks that have been clearly identified have been drawn in the sketch.

CT-32/La Balsa trackiste:

The presence of possible *Deltapodus* tracks in CT-32 has been previously noted ([S4-S5]). The site is preserved in the mudstone and peloidal limestone facies of Campos-Soto et al.[S2] and the tracks are preserved as concave epireliefs. One pes (CT-32-1p) shows a typical *Deltapodus* morphology. It is subrectangular (Fig.S5A) and small in sized with dimensions similar to those of the holotype of *D. ibericus*, FL = 0.44, FW = 0.28, and FL/FW ratio 1.57. Although the MP values are not very high, there is evidence of three rounded impressions in the anterior part representing the rounded ungual impressions. This footprint has considerable depth, which is the explanation for the poorly preserved ungual impressions. One of the lateral digits is more developed than the other so it is interpreted as a right footprint.

CT-64:

Campos-Soto et al.[S2] noted the presence of *Deltapodus*-like footprints in this small outcrop. Three footprints (Fig. S5B, C) can be identified; they are preserved in the non-channelized sandstone facies of Campos-Soto et al.[S2]. They are preserved as convex epireliefs in a sandstone layer, with the footprints being raised pedestals. A manus-pes set (Fig.S5B) and an isolated pes (Fig.S5C) can be identified. Both pes have a reversed elongated delta morphology with a clear rounded central digit. Both pes are small-sized and have the same FL (0.35 m), with a slight variations in FW (0.22-0.25) and the FL/FW ratio (1.4-1.59) because CT-64-1p is more elongated than CT-64-2p. The former track also shows a quadrangular posterior morphology in the posterior part of the footprint. CT-64-1m is not well-preserved and has a semicircular morphology. The heteropody is medium.

LPV-1-3R:

This locality has recently yielded some dinosaur tridactyl tracks[S6]. In a recent survey, a block containing 4 possible footprints have been discovered (Fig. S5D). Among them, a pes (Fig. S5E) with high morphological preservation stands out. The pes (LPV-1-3R-1p) is tridactyl, blunt-toed, elongate, and subtriangular (reversed delta) in shape. It is medium-sized, it has 0.42 m in length, and 0.26 m in width, with a FL/FW ratio of 1.61. In the anterior area, it only shows two clear blunt impressions characterized by very rounded hoof-like marks, presumably of digits II and III, whereas digit IV is poorly

impressed. LPV-1-3R-1m is interpreted as a possible manus print, although it is considerably shallower than the pes print. It is kidney-shaped and wider than long and it is located in a similar position to the manus-pes sets of the *D. ibericus* trackways. LPV-1-3R-2 is a partial footprint of difficult interpretation. This may represent a manus track heading in the opposite direction as LPV-1-3R-1 since it is kidney-shaped and wider than long. It shows evidence of striae. LPV-1-3R-3 is also a partially preserved track that is difficult to interpret, either as a manus print heading in the same direction as LPV-1-3R-1 or as a broken pes print where only the heel pad impression can be seen.

FA-11:

It is a new site where an isolated pes cast (Fig.S5F) preserved in sandstone as a natural cast (convex hyporelief) has been recovered. MAP-8430 is a deep cast, and digit impressions located in an anterior position can be recognized but not very clearly in the palmar view. This is a tiny specimen with FL = 0.23, FW = 0.16 and FL/FW ratio = 1.53.

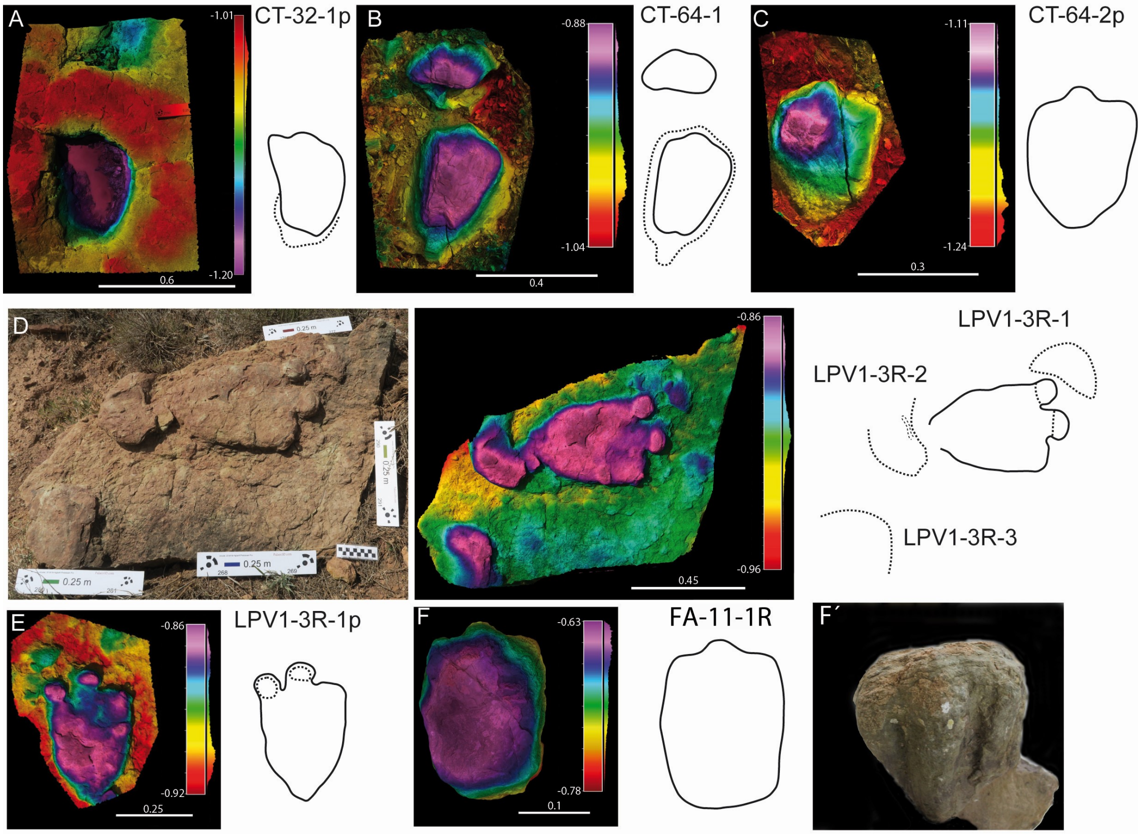

Fig. S5: False color-depth map of a selection of Deltapodus *tracks from different tracksites in the Maestrazgo Basin. A) CT-32-1p. B) CT-64-1. C) CT-64-2p. D) LPV-1-*

3R block. E) LPV-1-3R-1p. F) FA-11-1R (MAP-8430). F′) Picture of FA-11-1R (MAP-8430) in anterior view.

Barranco del Agua (BDA) tracksite:

This site was reported by Herrero-Gascón and Pérez-Lorente[S7] who described several manus and pes casts (BDA1-BDA10) assigned to *Deltapodus*. The authors provided a wealth of information regarding the plantar (sole) features of *Deltapodus* pes tracks such as a "slight medial concavity of the sole", a rounded heel, a stronger and more advanced projection of DII, and no separate digit prints or digital pads. The authors described the manus prints as crescent-shaped but showing variability from plano-convex to a concavo-convex morphology as a consequence of the autopod movement in the substrate. A review of the tracksite has been carried out. Among the tracks stand out block BDA2, which contains a partial trackway with two manus-pes sets (Fig. S6). BDA2-1p is partially broken, and the posterior part is poorly-preserved. BDA2-1m is well-preserved and semicircular in shape, showing a higher FL/FW ratio (0.71) than other manus tracks in the studied sample. BDA2-2p is medium-sized (FL= 0.36 and FW = 0.25) and is well impressed. The proportions of this pes are less than those of the holotype of *D.ibericus* (FL/FW ratio = 1.44). BDA2-2m is poorly impressed but characterized by a rounded morphology and a possibly high FL/FW ratio. The heteropody is low-medium (HI = 35). BDA-9 (MAP-8446) is an isolated manus cast that is kidney-shaped and has a considerable concavity (Fig. S6D). The variations in the FL/FW ratio in the manus prints at this site from values close to 0.7-0.8 to 0.4 in this latter specimen are noteworthy.

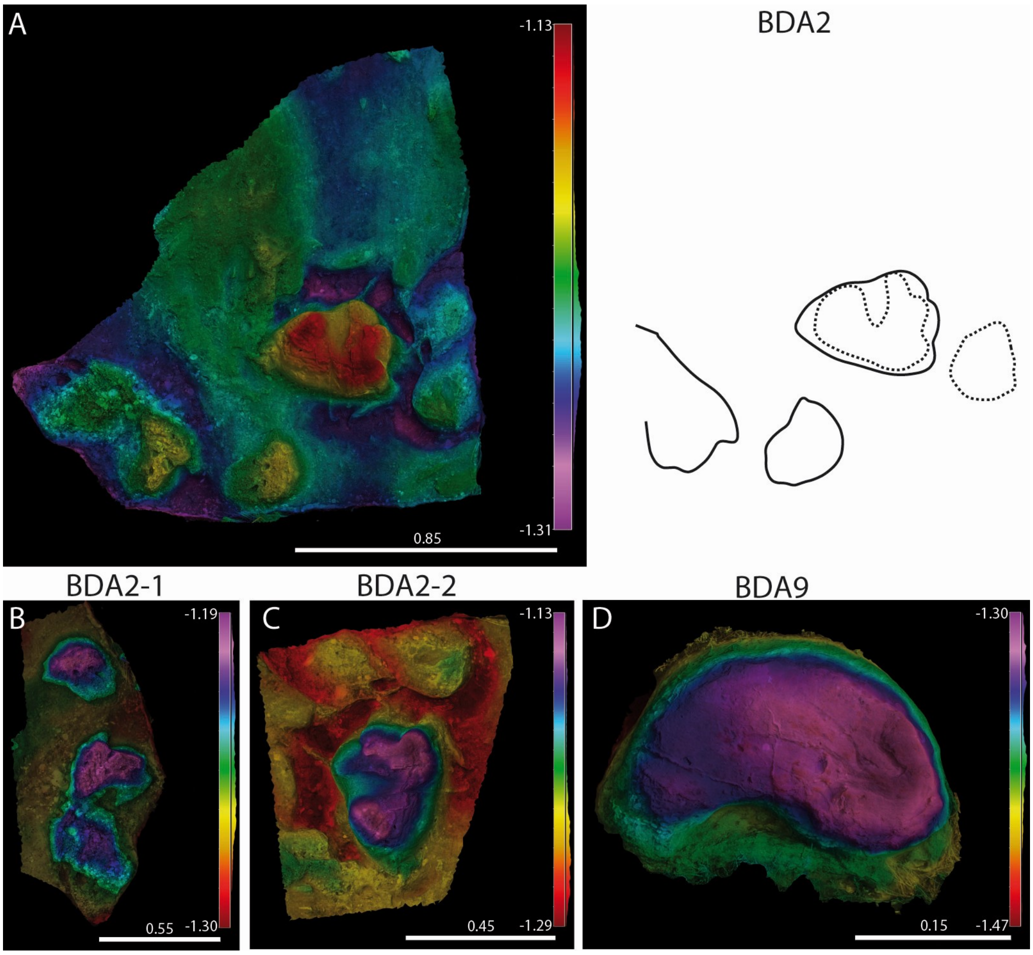

Fig. S6: Tracks from the Barranco del Agua tracksite. A) False color-depth map of BDA2, a partial trackway. B) False color-depth map of the BDA2-1 manus-pes set. C) False color-depth map of the BDA2-2 manus-pes set. D) False color-depth map of manus BDA9 (MAP-8446).

Aguilar 3 (AG-3)-El Rompido:

This site represents the second largest sample of studied stegosaur footprints. Interestingly, the site is preserved at the base of a nearly vertical calcareous (marly limestone) bed where the footprints are preserved as casts (convex hyporeliefs). On the surface of 12.5 m^2 more than 60 dinosaur footprints have been reported. Mampel et al., [S8] identified a total of 23 tracks (including the 8 manus-pes described here in detail) assigned to stegosaurs (*Deltapodus*-like) (Fig. 4 in the main text). In addition, the authors described almost 39 tridactyl tracks assigned to ornithopods. Almost at the same time, Herrero Gascón and Pérez-Lorente[S9] also described the tracksite providing a different interpretation of the trackmakers, considering them sauropods and theropods, respectively. Taken into account these different views, we provide here a new cartography (Fig. 4 in the main text) based on the data obtained from a 3D model built with a laser scan, and make special emphasis on the *Deltapodus*-like footprints (the tridactyl tracks will be described elsewhere). Eight manus-pes sets can be clearly identified whose configurations seem to represent 4 different trackways. Nonetheless, only T1 has 3 consecutive manus-pes sets. Generally, the pes prints are small in size (FL = 0.15-0.24 m) with a morphology that varies from subrectangular to reversed delta (subtriangular), with the anterior and middle parts being wider than the posterior parts. The digit impressions are not clearly visible in all the tracks, but when clearly preserved (e.g., AG3-2p, AG3-4p, AG3-9p) they are short and oriented in an anterior position, and DIII is slightly longer than DII, which is also slightly longer than DIV. AG3-9p shows evidence of clear blunt marks only in DII and DIII. Pes are quite elongated, although the FL/FW ratio is highly variable (1.35-1.72). The manus prints are also quite variable in shape (from oval, semicircular to kidney-shaped). Some manus impressions (e.g. AG3-4m) show a small medially oriented indentation that could represent a small pollex mark impression. The manus-pes sets show the manus located anteriorly or anterolaterally and very close to the pes, sometimes being overstepped. Some manus prints are smaller in size because of the overstepping by the pes, and the manus-pes distance is considerably short or almost absent. The heteropody is also quite variable but biased because of overstepping, so in the manus-pes sets without deformation, it shows medium (1.3) to high values (1:5). The trackways are wide gauge, with a clear distance between the inner parts of the tracks. The footprint rotation is also variable, with some prints clearly outwardly rotated.

There are clear examples of overstepping of the ornithopods into the stegosaur footprints, suggesting that the thyreophors walked first. Herrero Gascón and Pérez-Lorente[S9] noted that the quadrupedal tracks are among the smallest sauropod tracks in Spain. Their interpretation as sauropod tracks was based on the description in the pes impressions of 3 to 4 claw impressions oriented laterally. We could not identify such claw impressions in either of the pes impressions. Instead, there is the example of AG3-4p that is overprinted by a tridactyl track in the anterior part of the pes giving the impression that both tracks could be the same but the 3D model (Fig. 3e in the main text) clearly shows that they are two different tracks (drew as a sauropod footprint with claw impressions by Herrero Gascón and Pérez-Lorente[S9]).

Trackway 1 is composed of three consecutive manus-pes sets (AG3-2, AG3-3, AG3-4) with a pace length of approximately 0.50 m and a stride length of 0.90 m. The estimated speed is 0.89 m/s (3.2 km/h). Trackway 2 is composed of two complete manus-pes (consecutive from the right side) and two incomplete manus-pes that are not consecutive. The stride length is 0.92 m, and the estimated speed is 0.83 m/s (2.98 km/h). Both trackways are heading to the NE and are completely parallel with a very short intertrackway space (less than a meter). In addition, there are two other lineations that might represent another two trackways that are also heading to the NE and subparallel to the others.

AB-1 Ababuj:

AB-1 is a historic tracksite that was one of the first sites described in the Maestrazgo Basin[S10]. The site has mainly yielded theropod and sauropod tracks, but there is also a trackway (2AB-1) with oval footprints that has been considered *Deltapodus*-like[S11]. These footprints have been considered as 2 manus-pes sets where the manus are overprinted by the pes. The footprints measure 0.49 m. Alcalá et al.[S11] noted the similarities between these tracks and some of the tracks described in CT-1. These footprints have been reviewed at the site and also in a LaserScan 3D model carried out in 2019. The color maps do not show good evidence of the presence of the blunt-toed tridactyl pes that unequivocally identify *Deltapodus* tracks. On the other hand, the excavation of the lower area of the tracksite has yielded new footprints, one of which might represent a *Deltpodus* pes track (Fig.S7). It is an elongated track with dimensions of FL = 0.38 and FW = 0.25 and a FL/FW ratio of 1.52, which are within the range or

close to that of the holotype of *D. ibericus*. The anterior part of the footprint is slightly eroded and distorted by a large rim, but three blunt digits can be suspected.

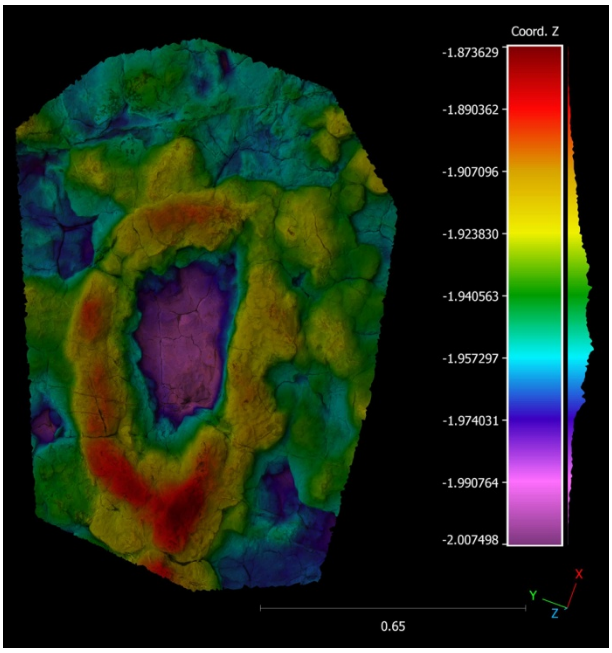

Fig. S7: False color-depth map of track AB1-1p

Visualization of morphometric data

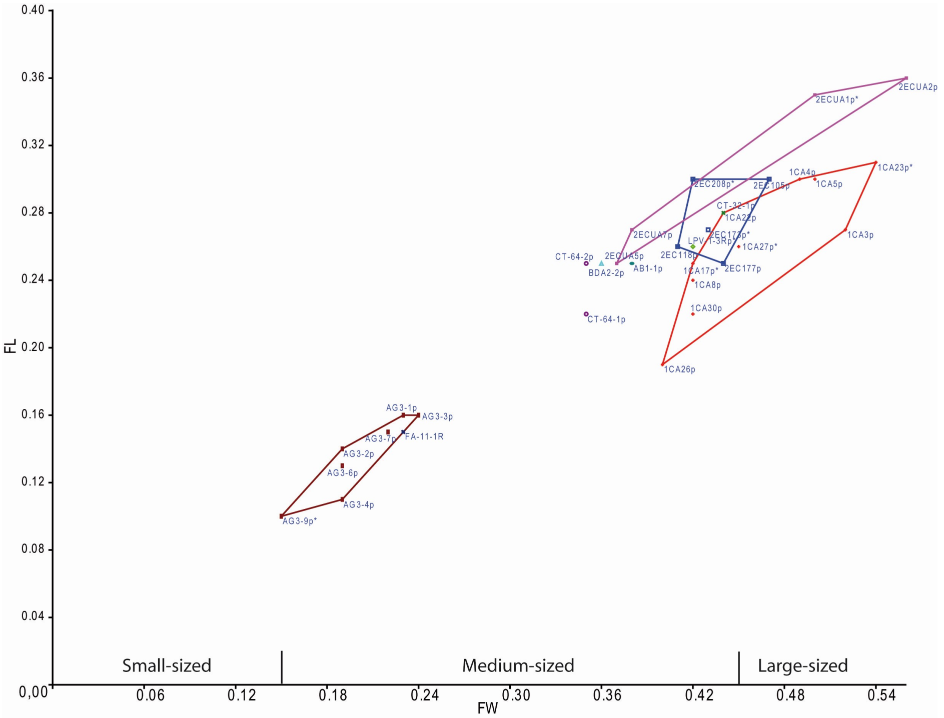

Fig. S8: Bivariate graph plotting the pes footprint length (FL) and footprint width (FW) of the studied sample.

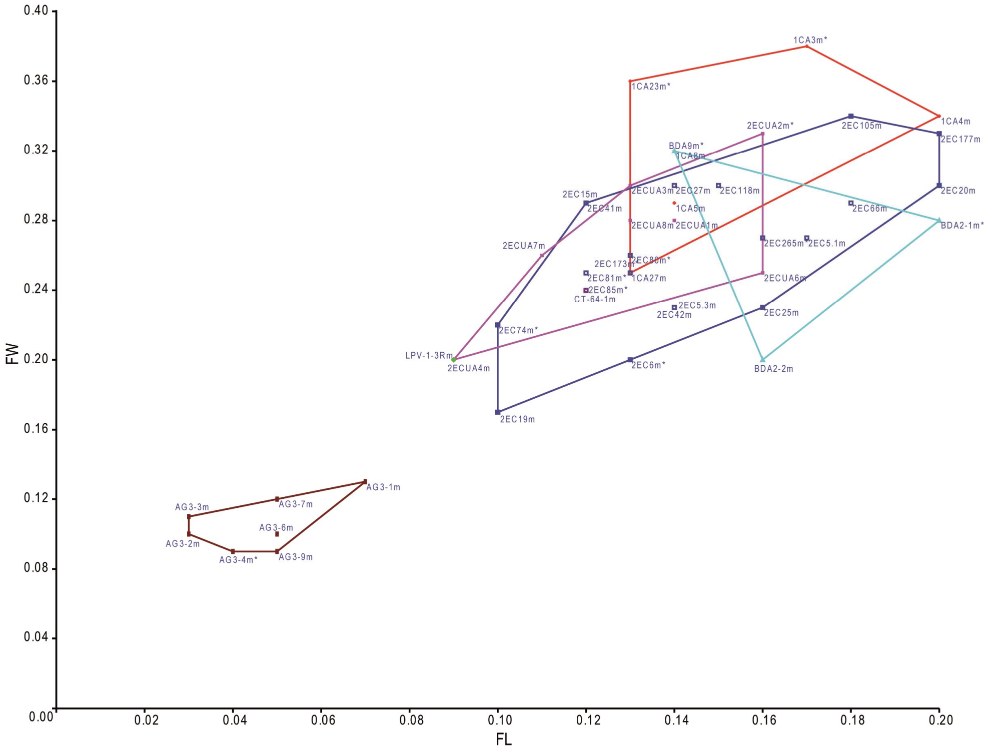

Fig. S9: Bivariate graph plotting the manus footprint length (FL) and footprint width (FW) of the studied sample.

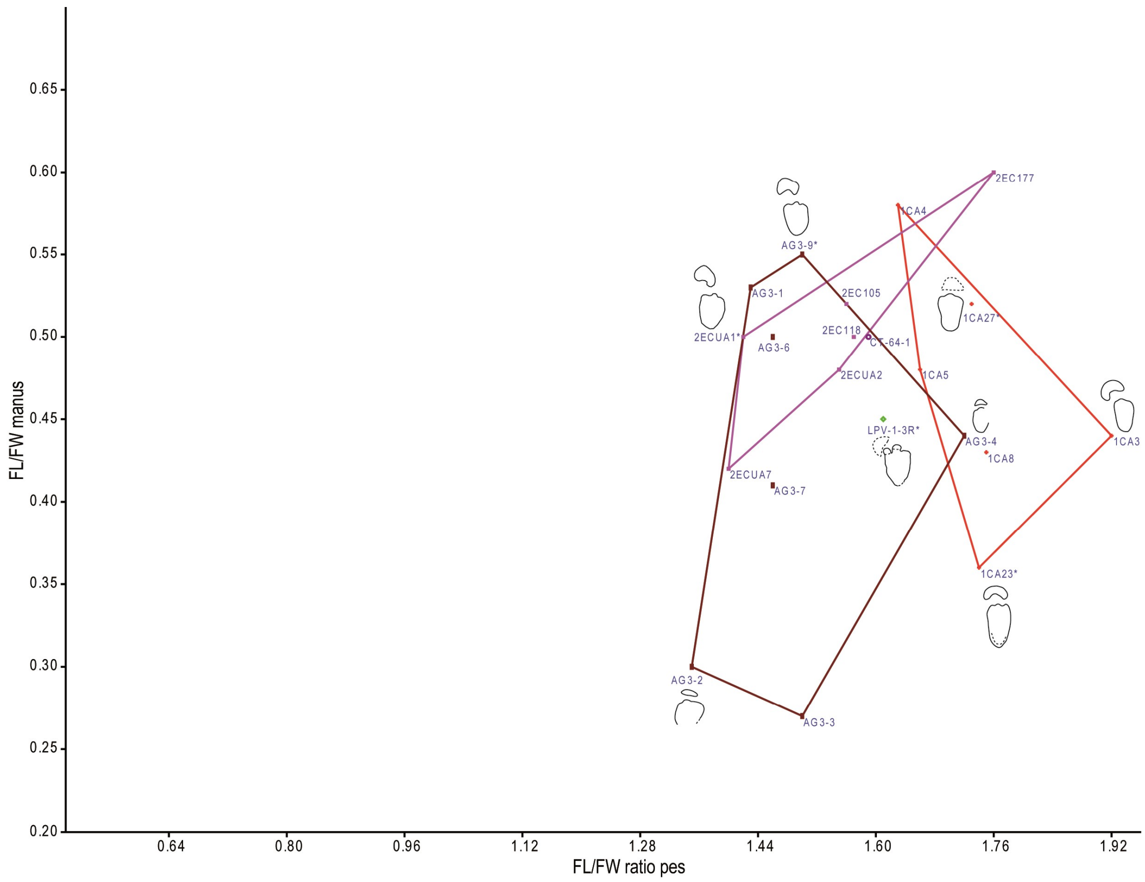

Fig. S10: Bivariate graph plotting the manus and pes footprint length/width ratio (FL/FW). Note that the main differences are in the elongation of the pes prints (X axis) and the manus print morphology (Y axis). Outline drawings not to scale.

Materials and methods:

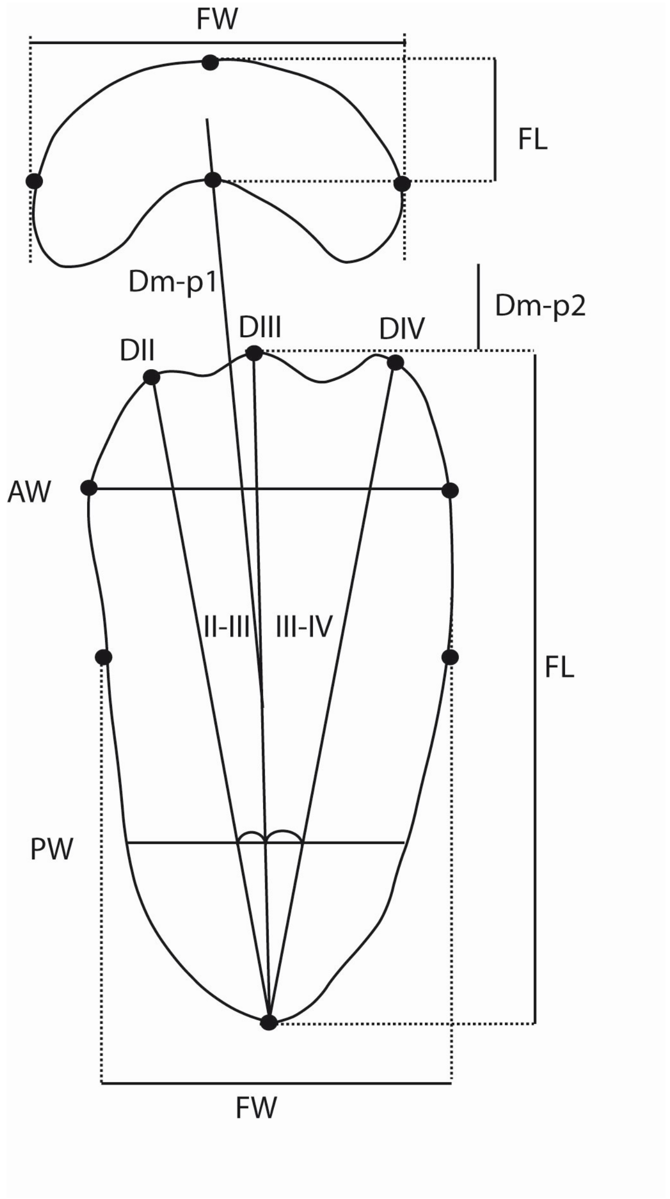

Fig. S11: Measurements taken for each studied individual footprint. FL, Footprint Length; FW, Footprint Width; DII, DIII, DIV, Digit II, III and IV lengths, respectively; AW, Anterior Width, PW, Posterior Width. II-III and III-IV, Divarication angles; D-mp, manus-pes distance. Dm-p1, manus-pes distance measured from the middle of the tracks; Dm-p2 manus-pes distance measured from the posterior part of the manus to the anterior part of the pes. The black dots are the landmarks used in the DigTrace software.

REFERENCES:

[S1] Alcalá, L., Pérez-Lorente, F., Luque, L., Cobos, A., Royo-Torres, R., & Mampel, L. (2014). Preservation of dinosaur footprints in shallow intertidal deposits of the Jurassic-Cretaceous transition in the Iberian Range (Teruel, Spain). Ichnos, 21(1), 19-31.

[S2] Campos-Soto, S., Cobos, A., Caus, E., Benito, M. I., Fernández-Labrador, L., Suarez-Gonzalez, P., ... & Alcalá, L. (2017). Jurassic Coastal Park: a great diversity of palaeoenvironments for the dinosaurs of the Villar del Arzobispo Formation (Teruel, eastern Spain). Palaeogeography, Palaeoclimatology, Palaeoecology, 485, 154-177.

[S3] Cobos, A., Royo-Torres, R., Luque, L., Alcalá, L., & Mampel, L. (2010). An Iberian stegosaurs paradise: The Villar del Arzobispo Formation (Tithonian–Berriasian) in Teruel (Spain). Palaeogeography, Palaeoclimatology, Palaeoecology, 293(1-2), 223-236.

[S4] Cobos , A., Alcalá, L., & Mampel, L. (2012). Stegosaurian footprints from the Jurassic-Cretaceous transition. In: The 11th Symposium of Mesozoic Terrestrial Ecosystems. Abstract book. 407-408.

[S5] Cobos, A., Royo-Torres, R., Alcalá, L., Luque, L., & Aberasturi, A. (2008). Nuevos datos de las icnitas de dinosaurios en la Formación Villar del Arzobispo (Teruel). XXIV Jornadas de la Sociedad Española de Paleontología, Colunga (Asturias, España), Libro de Resúmenes, pp. 25-26.

[S6] Guarido, A., Castanera D., Cobos, A. (2024).Icnitas de dinosaurios en el Jurásico Superior de La Puebla de Valverde (Teruel, España). Geogaceta, 75, 51-54.

[S7] Herrero Gascón, J., & Pérez Lorente, F. (2017). Hoof-like unguals, skin, and foot movements deduced from *Deltapodus* casts of the Galve Basin (Upper Jurassic-Lower Cretaceous, Teruel, Spain). Ichnos, 24(2), 146-161.

[S8] Mampel, L., Cobos, A., Alcalá, L., Espílez, E., Royo-Torres, R., González, A., & Gascó, F. (2011). Icnitas de dinosaurios en Aguilar del Alfambra (Teruel, España). Teruel, 93(1), 41-54.

[S9] Herrero Gascón, J., & Pérez Lorente, F. (2012). El Rompido (Aguilar del Alfambra). Icnitas de dinosaurios en la Formación Villar del Arzobispo. Teruel. Geogaceta, 51, 39-42.

[S10] Alcalá, L., & Martín Escorza, C. (1995). Huellas de dinosaurios en el Jurásico Superior de Ababuj (Teruel). Geogaceta, 17, 19-22.

[S11] Alcalá, L., Cobos, A., Espílez, E., Gascó, F., Mampel, L., Martín Escorza, C., & Royo-Torres, R. (2012). Icnitas de dinosaurios en la Formación Villar del Arzobispo de Ababuj (Teruel, España). Geogaceta, 51, 35-38.
